# Multiple evolutionary origins of *Trypanosoma evansi* in Kenya

**DOI:** 10.1371/journal.pntd.0005895

**Published:** 2017-09-07

**Authors:** Christine M. Kamidi, Norah P. Saarman, Kirstin Dion, Paul O. Mireji, Collins Ouma, Grace Murilla, Serap Aksoy, Achim Schnaufer, Adalgisa Caccone

**Affiliations:** 1 Biotechnology Research Institute, Kenya Agricultural and Livestock Research Organization, Kikuyu, Kenya; 2 Department of Biomedical Sciences and Technology, School of Public Health and Community Development, Maseno University, Maseno, Kenya; 3 Yale School of Public Health, Department of Epidemiology of Microbial Diseases, New Haven, CT, United States of America; 4 Department of Ecology & Evolutionary Biology, Yale University, New Haven, CT, United States of America; 5 Centre for Geographic Medicine Research Coast, Kenya Medical Research Institute, Kilifi, Kenya; 6 Centre for Immunity, Infection & Evolution, and Institute of Immunology & Infection Research, University of Edinburgh, Edinburgh, Scotland, United Kingdom; International Centre of Insect Physiology and Ecology, KENYA

## Abstract

*Trypanosoma evansi* is the parasite causing surra, a form of trypanosomiasis in camels and other livestock, and a serious economic burden in Kenya and many other parts of the world. *Trypanosoma evansi* transmission can be sustained mechanically by tabanid and Stomoxys biting flies, whereas the closely related African trypanosomes *T*. *brucei brucei* and *T*. *b*. *rhodesiense* require cyclical development in tsetse flies (genus *Glossina*) for transmission. In this study, we investigated the evolutionary origins of *T*. *evansi*. We used 15 polymorphic microsatellites to quantify levels and patterns of genetic diversity among 41 *T*. *evansi* isolates and 66 isolates of *T*. *b*. *brucei* (n = 51) and *T*. *b*. *rhodesiense* (n = 15), including many from Kenya, a region where *T*. *evansi* may have evolved from *T*. *brucei*. We found that *T*. *evansi* strains belong to at least two distinct *T*. *brucei* genetic units and contain genetic diversity that is similar to that in *T*. *brucei* strains. Results indicated that the 41 *T*. *evansi* isolates originated from multiple *T*. *brucei* strains from different genetic backgrounds, implying independent origins of *T*. *evansi* from *T*. *brucei* strains. This surprising finding further suggested that the acquisition of the ability of *T*. *evansi* to be transmitted mechanically, and thus the ability to escape the obligate link with the African tsetse fly vector, has occurred repeatedly. These findings, if confirmed, have epidemiological implications, as *T*. *brucei* strains from different genetic backgrounds can become either causative agents of a dangerous, cosmopolitan livestock disease or of a lethal human disease, like for *T*. *b*. *rhodesiense*.

## Author summary

*Trypanosoma evansi* is an important pathogen of the camel and other livestock where it is a causative agent of surra (an economically burdensome disease). The *T*. *evansi* is found in Kenya and the rest of the world. This study indicates that *T*. *evansi* originated recently from multiple *Trypanosoma brucei* strains from different genetic backgrounds. This suggests multiple independent evolutionary origins of some complex traits that may have facilitated mechanical transmission in *T*. *evansi* and subsequently enabled the parasite to escape the obligate link with the African tsetse fly vector. This evolutionary origin appears to have occurred repeatedly. Our results provide a more comprehensive understanding of the epidemiology of surra, provide recommendations for future work, and indicate a need to consider the risk of horizontal transfer of epidemiologically relevant traits among different *Trypanosoma* genetic backgrounds in any control campaign. Thus, our study is an important contribution to the field, and represents an important step towards the ultimate aim of trypanosomiasis prevention and/or elimination.

## Introduction

*Trypanosoma evansi* is an important disease-causing parasite of livestock in many African, Asian and South American countries. *T*. *evansi* belongs to a group of five closely related named taxa of various ranks found in a wide diversity of mammalian hosts; *Trypanosoma brucei brucei*, *T*. *b*. *gambiense*, *T*. *b*. *rhodesiense*, *T*. *evansi*, and *T*. *equiperdum* [1–5]. Three of these closely related parasites (*T*. *b*. *brucei*, *T*. *b*. *gambiense*, and *T*. *b*. *rhodesiense*) are only found in sub-Saharan Africa, where they require transmission by a tsetse fly vector and cause nagana in animals and sleeping sickness in humans, respectively [6,7]. The other two members of this group (*T*. *evansi* and *T*. *equiperdum*) are found both inside and outside the African continent, use other means of transmission, and are responsible for surra in wild and domestic animals [8] and dourine in equines [9], respectively.

The formal taxonomy of this group of closely related trypanosomes is in flux and currently reflects their disease outcome and means of transmission rather than their evolutionary relationships [10–14]. For example, strains of the human infective named subspecies, *T*. *b*. *rhodesiense*, are genetically closer to different *T*. *b*. *brucei* strains than to other strains from the same named subspecies [3,13,14,15]. Similarly, the taxonomic rank of *T*. *evansi* and *T*. *equiperdum* is in question because the few *T*. *evansi* and *T*. *equiperdum* strains that have been analyzed to date are genetically closer to different *T*. *b*. *brucei* strains than to other strains from the same named species [1,10,11,12,13,14,16,17]. This indicates that neither named species is monophyletic and suggests multiple origins from *T*. *b*. *brucei*. Despite the clear need for taxonomic revisions, and to avoid confusion, we use the established nomenclature. We further classify *T*. *evansi* based on their mitochondrial DNA (kinetoplast DNA or kDNA) configuration of type A or B [18–20] and their antigenic variant surface glycoprotein (VSG) Rode Trypanozoon antigenic type (RoTat) 1.2, used in serological and PCR-based diagnostic tests [11,21,22,23,24].

*Trypanosoma evansi* is the most geographically widespread of these trypanosomes [2,25], and some authors have suggested that it originated in camels in Africa [8,12], where it occurs in all countries where these animals are found. This distribution extends along a northern line from Senegal to Mauritania, Morocco, Algeria, Tunisia, Libya, Egypt, Sudan, Eritrea, and Ethiopia, and the northern parts of Mali, Burkina Faso, Niger, Nigeria, Chad, Somalia, and Kenya [8]. Outside of Africa, *T*. *evansi* is thought to be limited by dispersal routes rather than the presence of camels and occurs in Asia and South America [2]. Both inside and outside of Africa, surra affects a variety of animals besides camels, including horses, cattle, buffalos, small ruminants, and dogs [2,26], causing thousands of animal deaths per year. Although the net economic losses attributable to *T*. *evansi* infections are difficult to estimate [2,26], mortality rates of animals affected and total effort invested in chemotherapeutic interventions indicate significant economic losses and social impacts among regions of the world [5,27,28,29].

African trypanosomes within the *T*. *brucei* complex require cyclical development within the tsetse fly vector to complete their life cycle and transmission [30,31]. In contrast, *T*. *evansi* and *T*. *equiperdum* exist exclusively as developmental forms equivalent to the bloodstream form of *T*. *brucei*. *T*. *evansi* is transmitted mechanically by biting insects or, in South America, alternatively by vampire bats [26]. *T*. *equiperdum* is transmitted sexually during intercourse in horses [9]. Tsetse-independent transmission enabled these parasites to move out of the tsetse fly belt in sub-Saharan Africa. Mechanical transmission is a non-specific process that can take place when a vector undergoes interrupted feeding between hosts. Although any biting insect could transmit *T*. *evansi* from one host to the next, the insects responsible for most of its transmissions are haematophagous insects, such as horseflies and stable flies [32].

In addition to their ability to bypass the tsetse fly vector, all *T*. *evansi* (and *T*. *equiperdum*) strains analyzed so far are also characterized by having no or dysfunctional kinetoplast DNA, a trait referred to as dyskinetoplastidy [10,16,18,33]. Where present, kDNA has suffered homogenization of the minicircle component, which consists of more than 200 distinct classes in a tsetse transmission competent strain of *T*. *brucei* [34]. In all *T*. *evansi* strains analyzed to date, kDNA is dominated by either type A or type B minicircles [10,18,22,35]. Minicircle heterogeneity is essential for mitochondrial gene expression in trypanosomes [25]. As a consequence of its dyskinetoplastidy, *T*. *evansi* can therefore no longer complete cyclical development in the tsetse fly, and this could be one of the driving forces for the switch to mechanical transmission [16,18,36]. Another consequence of their inability to complete their development in tsetse flies is that both *T*. *evansi* and *T*. *equiperdum* strains do not undergo sexual reproduction. Although these peculiarities unite all *T*. *evansi* (and *T*. *equiperdum*) strains, there is significant variation in other traits such as virulence among parasite strains and animal host species [37].

**Fig 1:**
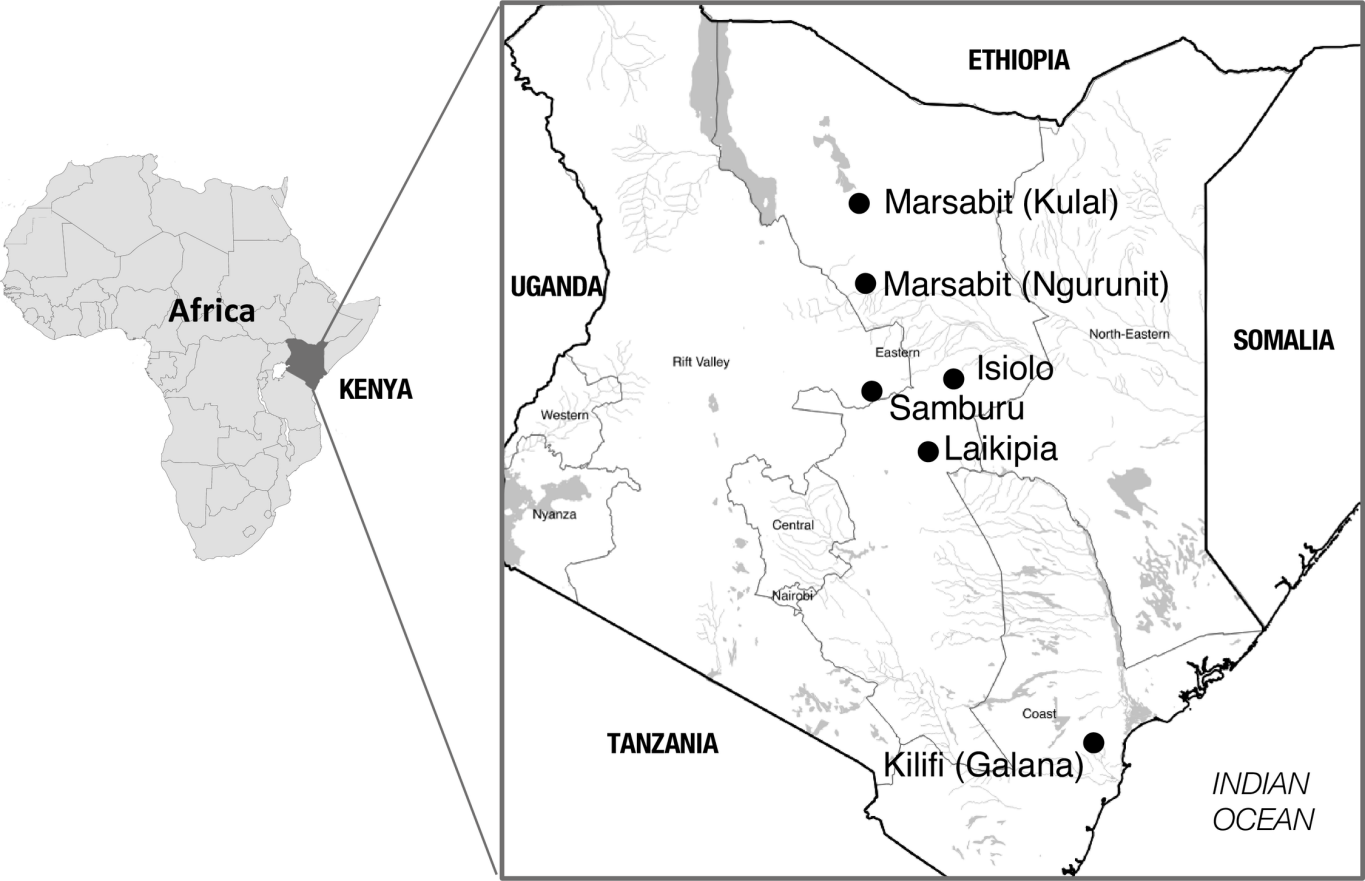
Map of Africa showing in black location of Kenya (https://commons.wikimedia/wiki/Atlas_of_the_world). The insert to the right shows the location of the *Trypanosoma evansi* (Tev) and *T*. *brucei brucei* (Tbb) isolates genotyped for this study (small black circles). Sample details are listed in Table 1.

In this study, we screened for genetic variation at a set of 15 highly variable polymorphic loci in a group of 35 *T*. *evansi* isolates from Kenya (Fig 1, Table 1). In this area both *T*. *evansi* and *T*. *brucei* co-occur, making it a potential area where the trypanosome host shift into camels might have occurred [38]. The climate of this region is semi-arid and supports husbandry of both camels, the typical host of *T*. *evansi* in this region, and cattle and goats [2], common hosts of *T*. *brucei*. The goal of this paper is to quantify levels and patterns of inter-strains genetic diversity among to understand the evolutionary origin of different *T*. *evansi* strains. This will help control and monitor disease spread by providing data that inform on the rate and modality of novel genotypic combinations that exists in the circulating *T*. *evansi* strains. Furthermore, this data provides general insights on the different ways *T*. *brucei* strains can evolve into epidemiologically novel parasites despite their very similar genetic background. This general phenomenon has important epidemiological implications for both the animal and human diseases that they cause.

**Table 1:** Sample details and PCR assay results of *T*. *evansi* genotyped for this study showing sample ID, isolate source and reference in footnote, kinetoplast DNA (kDNA) type, PCR assay results (ITS1 + indicates pathogenic African trypanosome, SRA–indicates not *T*. *b*. *rhodesiense*, RoTat 1.2 + indicates the serological diagnostic antigen variant, A281del + indicates deletion of a GTC (Ala) triplet in F_O_F_1_-ATPase subunit γ unique to *T*. *evansi* isolates of kDNA type A, n/a indicates failure of the positive PCR control), host of isolation, the locality of origin and year of isolation. See also S1 Table for isolates genotyped in previous studies.

A.			PCR assay							
Sample ID	Isolate ^source^ [reference]	kDNA type	ITS 1	SRA		Ro Tat 1.2	A 281del	Host	County (town) / Country	Year of isolation
K2469	KETRI2469 ^a^	A†	+		-	-	+	Camel	Marsabit (Kulal)	1979
K2444	KETRI2444 ^a^	A†	+		-	-	+	Camel	Marsabit (Kulal)	1979
K2467	KETRI2467 ^a^	A†	+		-	-	+	Camel	Samburu	1979
K3789	KETRI3789 ^a^	A†	+		-	-	+	Camel	Samburu	2003
K3793 ^#^	KETRI3793 ^a^	Unkn.	+		-	-	n/a	Camel	Laikipia	1995
K3930	KETRI3930 ^a^	A†	+		-	-	+	Camel	Samburu	2003
K3931	KETRI3931 ^a^	A†	+		-	-	+	Camel	Marsabit (Kulal)	2003
K2443	KETRI2443 ^a^ [84]	A	+		-	-	+	Camel	Marsabit (Kulal)	1979
K2450 ^#^	KETRI2450 ^a^ [84]	Unkn.	+		-	-	n/a	Camel	Kilifi (Galana)	1979
K2455	KETRI2455 ^a^ [84]	A†	+		-	-	+	Camel	Kilifi (Galana)	1979
K2458	KETRI2458 ^a^ [84]	A†	+		-	-	+	Camel	Kilifi (Galana)	1979
K2465	KETRI2465 ^a^ [84]	A†	+		-	-	+	Camel	Marsabit (Kulal)	1979
K2466	KETRI2466 ^a^ [84]	A†	+		-	-	+	Camel	Marsabit (Kulal)	1979
K2470	KETRI2470 ^a^ [84]	A†	+		-	-	+	Camel	Marsabit (Kulal)	1979
K2439	KETRI2439 ^a^ [84]	A	+		-	+	+	Camel	Marsabit (Kulal)	1979
K2441	KETRI2441 ^a^ [84]	A†	+		-	+	+	Camel	Marsabit (Kulal)	1979
K2442	KETRI2442 ^a^ [84]	A†	+		-	+	+	Camel	Marsabit (Kulal)	1979
K2446	KETRI2446 ^a^	A†	+		-	+	+	Camel	Marsabit (Kulal)	1979
K2449	KETRI2449 ^a^	A†	+		-	+	+	Camel	Kilifi (Galana)	1979
K2451	KETRI2451 ^a^ [84]	A†	+		-	+	+	Camel	Kilifi (Galana)	1979
K2453	KETRI2453 ^a^ [84]	A†	+		-	+	+	Camel	Marsabit (Kulal)	1979
K2454	KETRI2454 ^a^ [84]	A	+		-	+	+	Camel	Marsabit (Kulal)	1979
K2456	KETRI2456 ^a^ [41]	A	+		-	+	+	Camel	Kilifi (Galana)	1979
K2457	KETRI2457 ^a^ [84]	A†	+		-	+	+	Camel	Marsabit (Kulal)	1979
K2479**	KETRI2479 ^a^ [18,19,84,85]	B	+		-	-	-	Camel	Marsabit (Ngurunit)	1979
K2481	KETRI2481 ^a^ [84]	A†	+		-	+	+	Camel	Marsabit (Kulal)	1979
K3548	KETRI3548 ^a^ [84]	A†	+		-	+	+	Camel	Isiolo	1994
K3550	KETRI3550 ^a^ [84]	A†	+		-	+	+	Camel	Isiolo	1994
K3551	KETRI3551 ^a^ [84]	A†	+		-	+	+	Camel	Isiolo	1994
K3552	KETRI3552 ^a^ [84]	Non-A/B	+		-	+	-	Camel	Isiolo	1994
K3553	KETRI3553 ^a^	A†	+		-	+	+	Camel	Isiolo	1994
K3556	KETRI3556 ^a^ [84]	A†	+		-	+	+	Camel	Isiolo	1994
K3557	KETRI3557 ^a^ [84]	Non-A/B	+		-	+	-	Camel	Isiolo	1994
K3558	KETRI3558 ^a^ [84]	A†	+		-	+	+	Camel	Isiolo	1994
K3576*	KETRI3576 ^a^ [84]	Unkn.	+		-	+	n/a	Camel	Marsabit (Ngurunit)	1994
STIB810	STIB810 ^b^ [36]	A	+		-	+	n/a	Buffalo	China	1985
C13	C13 [19]	A	+		-	+	n/a	Camel	Kenya	1981

^#^ *T*. *evansi* assignment based on camel host alone

^†^ kDNA type based on A281del PCR assay alone

^a^ Kenya Trypanosomiasis Research Institute

^b^ Swiss Tropical Institute Basel

** high virulence

* low virulence

## Materials and methods

### Trypanosome isolates

For the purpose of this work, and in line with microbiological convention, we have defined the terms isolates and strains as follows. An isolate was obtained by sampling a particular animal at a particular point in time. A strain is an isolate or group of isolates that can be distinguished from other isolates by phenotypic and or genotypic characterization [39]. We analyzed a total of 41 *T*. *evansi* isolates. The majority of these isolates are from Kenya (Fig 1) and currently stored at the KETRI cryobank [40] at KALRO-BRI (Kikuyu, Kenya). These samples had been collected at several time points and some had previously been classified as *T*. *evansi* based on host species (camel vs. non-camel), region of isolation, and kDNA minicircle type (Table 1). The virulence of two of these isolates, K2479 [19,41] and K3576, were experimentally characterized in mice, based on relative levels of parasitemia and host survivorship in infected mice (Kamidi et al., in prep). The remaining *T*. *evansi* isolates came from multiple sources (Table 1, S1 Table) and have been well-characterized in past studies and, in some cases, were part of recent genetic studies [10,15,18,19,36].

To provide a spatial breadth to our study and to be able to connect it with previous microsatellite analyses we also included 66 *T*. *b*. *brucei* and *T*. *b*. *rhodesiense* isolates (S1 Table) from across sub-Saharan Africa that have also been extensively characterized [10,15,42]. These isolates included at least one representative from each of the genetic clusters previously identified in sub-Saharan Africa [15]. Thus, the final sample set consisted of 107 *T*. *brucei* and *T*. *evansi* isolates, including 4 from buffalo in Asia and 103 from a variety of mammalian hosts in Africa, with a special focus on isolates from camels (Fig 1, Table 1) and wildlife (S1 Table) in Kenya.

### DNA extractions and PCR based diagnostic tests

DNA was extracted from isolates that did not have DNA available using either the Qiagen DNeasy Blood and Tissue Kit (Qiagen, Germany), following manufacturer’s protocols, or a phenol and chloroform protocol for samples for which DNA extractions were already available [43]. To further classify presumptive *T*. *evansi* samples not previously well classified [18,19,36]; we carried out a set of four diagnostic PCR tests for 37 isolates including 34 isolates for which we did not have certain classification (Table 1). First, we used PCR amplification of a 480 bp fragment of the Internal Transcribed Spacer (ITS1) of the ribosomal DNA[44], to confirm all isolates were pathogenic African trypanosomes. We then used PCR amplification of a 284 bp fragment of the serum resistance-associated (SRA) gene [45], to confirm isolates were not *T*. *b*. *rhodesiense*.

Then, we performed a PCR assay to identify isolates with the VSG antigen type RoTat 1.2, used in serological and PCR-based diagnosis, that targets a 488 bp fragment of the RoTat 1.2 variant, as per previous protocol [23]. Although this gene occurs in most *T*. *evansi* type A [23,46], it has been reported that *T*. *evansi* type B and some *T*. *evansi* type A strains may not have it [46,47]. In addition, *T*. *evansi* strains can lose the kinetoplast entirely [10,16] which would lead to a false negative result in a diagnostic PCR assay for type A minicircles. Thus, as an alternative to identify type A *T*. *evansi*, we designed a novel PCR assay. This assay targets a 3-bp deletion (GTC codon, corresponding to alanine 281) in the nuclear encoded subunit **γ** (systematic TriTrypDB ID Tb927.10.180) of the mitochondrial F_O_F_1_-ATPase. This deletion is unique to all *T*. *evansi* type A screened so far and to some closely related strains that had been classified as *T*. *equiperdum* [10]. This mutation is critical to compensate for loss of functional kinetoplast DNA in this group of *T*. *evansi/T*. *equiperdum* [48]. The assay consists of two PCR reactions, a diagnostic and a control PCR reaction (S1 Fig). The diagnostic reaction (using primer combination F1/R1) is designed to amplify an 855 bp fragment of F_O_F_1_-ATPase subunit γ, if at least one allele in the strain has this 3-bp deletion (named A281del). The control PCR reaction (using primer combination F1/R2) amplifies an 863 bp long fragment of the same region, regardless of kDNA type. Both PCR reactions were carried out in 10 μl volumes consisting of 5 μl 2X Type-It (Qiagen), 0.25 μM of each primer, 10 ng of genomic DNA and dH_2_O. A touchdown thermal cycling protocol included a 5 min initial denaturation at 95°C, 10 cycles touchdown (95°C for 30 sec, 50°C minus 1°C per cycle for 30 sec, and 72°C for 1 min), and 30 cycles amplification (95°C for 30 sec, 40°C for 30 sec, and 72°C for 1 min), followed by a 7 min final extension period. All PCR runs included the isolates RoTat1.2 (OB106), a *T*. *evansi* type A, and cp24, a *T*. *b*. *brucei* from Balmer et al [15], as positive and negative controls, respectively (Table 1).

### Microsatellite genotyping

We used fifteen microsatellite loci extensively validated in previous studies and using the same previously published protocols[49,50]. Primer sequences for amplification and chromosomal locations of the loci can be found in S2 Table. Amplifications were performed with fluorescently labeled forward primers (6-FAM and HEX) using a standard PCR in 13 μl reaction volumes containing approximately 100 ng of genomic DNA, 5 μl of Type-it Master Mix (Qiagen, Germany) and 1 μl each of forward and reverse primers (10 μM starting concentration). PCR products were then multiplexed, combined with size standard (Applied Biosystems ROX500) and highly deionized formamide, and genotyped on an ABI 3730xl DNA Analyzer (Applied Biosystems Inc, USA) at the DNA Analysis Facility on Science Hill at Yale University (http://dna-analysis.yale.edu/)). Alleles were scored using the program GeneMarker v 2.4.0 (Soft Genetics, State College, PA, USA) with manual editing of the automatically scored peaks.

### Identification of distinct genetic clusters

To evaluate evolutionarily distinct genetic clusters within our dataset, we included all 107 *T*. *b*. *brucei*, *T*. *b*. *rhodesiense*, and *T*. *evansi* isolates in Bayesian cluster analyses using STRUCTURE *v2*.*3*.*4* [51]. STRUCTURE runs indicated a K value (number of clusters) of less than ten. Thus, we performed 20 runs with a burn-in of 5,000 and a total of 250,000 iterations to assess the optimal K value with the Evanno method [52], using the Clustering Markov Packager Across K (CLUMPAK) [53]. For final assignments of isolates to clusters, we performed 10 runs for K values one through ten with a burn in of 50,000 and 250,000 iterations. Each isolate was assessed for probability of assignment (Q) to each of the K clusters identified in the STRUCTURE analysis. We considered Q>0.80 as a “certain assignment”, and Q<0.80 as an “uncertain assignment". We further evaluated evolutionary relationships and the levels of genetic differentiation among and within *T*. *evansi* and *T*. *brucei* genetic clusters and isolates of uncertain assignment using principal components analysis (PCA) of microsatellite data in the “adegenet” package in R v3.0.2 (R Development Core Team). We estimated the centroid and region encompassing 95% of the variance observed within *T*. *brucei* subgroups identified in the STRUCTURE analysis.

### Estimating levels of genetic diversity and differentiation

In order to compare levels of genetic diversity and differentiation among *T*. *evansi* isolates with those found among *T*. *brucei* (*T*. *b*. *brucei* + *T*. *b*. *rhodesiense*) isolates, we estimated levels of diversity within the STRUCTURE defined clusters as well as levels of differentiation between and within clusters. For these analyses, we included only isolates with high probability of assignment (Q > 0.80) to STRUCTURE-based clusters at three levels: (i) all isolates regardless of taxonomy, (ii) *T*. *brucei* isolates only, and (iii) *T*. *evansi* isolates only.

To understand diversity within clusters at these three levels, we estimated allelic richness (A_R_) in FSTAT v1.2 [54], observed and expected heterozygosity (H_O_ and H_E_) and the related Fisher’s inbreeding coefficient (F_IS_) in the R package HIERFSTAT *v0*.*4–10* [55]. To understand patterns of within-cluster genetic distance at these three levels, we calculated pairwise genetic distance between isolates using the Reynolds distances [56]. We estimated a distance tree using the UPGMA method implemented in the “PopPR” v2.3.0 package [57,58] in R with 1000 bootstrap replicates. We then tested for significant differences in within-cluster genetic distances with an analysis of variance (ANOVA) followed by a Tukey-Kramer HSD test performed in JMP v11.2 (SAS Institute Inc., Cary, NC, USA, 1989–2012). To ensure that the time of isolation did not account for cluster assignment, we used the software JMP to perform a Chi-square test of the time of isolation (by decade), with the taxon of each sample included as a co-variate.

Finally, to understand patterns of among-cluster differentiation at the same three levels, we estimated pairwise F_ST_ in ARLEQUIN *v*.*3*.*5* [59] with Wright’s statistics [60], following the variance method [61], using 10,000 permutations, 1,000,000 Markov chain steps, and 10,000 dememorization steps to obtain exact p-values.

## Results and discussion

### PCR based diagnostic tests

Results from the PCR assays are presented in Table 1. We found that all of the KETRI isolates amplified in the PCR test that is diagnostic for the ITS1 region of all African trypanosomes considered pathogenic: Members of the subgenera *Nannomonas (T*. *congolense)*, *Duttonella (T*. *vivax)* and *Trypanozoon* (*T*. *brucei*, *T*. *evansi*, *T*. *equiperdum*) [22]. In contrast, *T*. *lewisi* and *T*. *theileri*, which are considered non-pathogenic but can be found in many areas of the world, including Kenya, have been reported to not give a positive signal, presumably because their ITS region is more divergent [22]. All isolates were also SRA negative, confirming the absence of *T*. *b*. *rhodesiense* isolates. For the A281del. PCR assay, five isolates could not be determined because they failed to amplify in the positive control reaction (n/a in Table 1). Of those that amplified, we found 29 isolates to be A281del positive, indicating that they are *T*. *evansi* type A, and 3 isolates that were A281del negative, indicating that they could be either type B or something else, but not type A. Only 20 of the isolates tested were positive for the RoTat 1.2 gene (including, as expected, STIB810 and C13), indicating a diversity of VSG antigen types in our dataset. Although it has been reported that type A *T*. *evansi* isolates are typically RoTat1.2 positive [10,21,24], we found that of the 29 A281del positive isolates, only 17 were RoTat 1.2 positive while 12 were RoTat 1.2 negative (Table 1). The combination of these PCR assays suggests that, at least in Kenya, *T*. *evansi* isolates that are type A but RoTat1.2 negative are more prevalent than expected [23,46,47], which could result in a considerable frequency of false negatives for current diagnostic tools for surra [23,24].

### Identification of distinct genetic clusters

**Fig 2:**
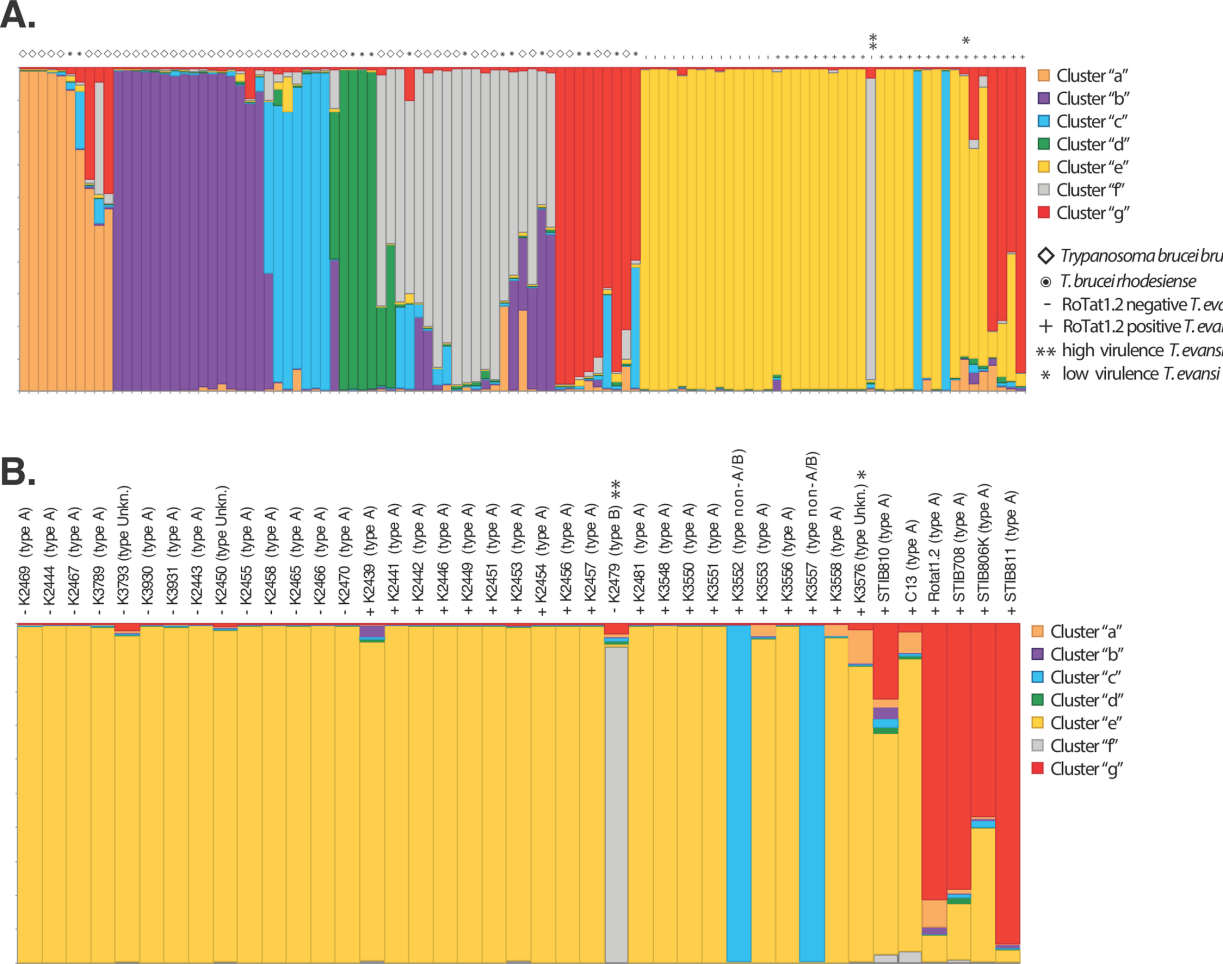
Plot of assignment scores of all isolates using STRUCTURE v2.3.4 [51] with K = 7 of **(A)** all isolates, and **(B)** the close up of *Trypanosoma evansi* isolates (Tev) with labels added showing isolate ID and kDNA type in parentheses (based on literature, where available, or predicted from the A281del PCR assay). Each vertical bar represents an isolate’s probability of assignment to one of seven genetic clusters "a" through "g" shown in orange, purple, blue, green, yellow, grey and red, as presented in the legend to the right. *T*. *brucei brucei* is indicated with a diamond, *T*. *b*. *rhodesiense* is indicated with a bullet point, and *T*. *evansi* is indicated by a plus "+" if RoTat 1.2 positive and minus "-" if RoTat 1.2 negative. The high virulence isolate is marked with a double asterix "**", and the low virulence isolate is marked by a single asterix "*". Note that Tev isolates in panel B are ordered according to Table 1 and not strictly according to cluster assignment.

The results suggested a K-value of 2, and thus the presence of two distinct genetic clusters, as the most likely hierarchical level of population structure that best fits the method’s assumptions (S2 Fig). One of these two clusters (S3 Fig; top panel, orange color) includes most but not all *T*. *evansi* isolates, while the other includes all of the *T*. *brucei brucei* and *T*. *b*. *rhodesiense* isolates (S3 Fig; top panel, blue color). The next best fit of K = 7 was able to distinguish structure within *T*. *brucei*, suggesting the presence of seven distinct genetic units. Assignment to these clusters for the 107 isolates analyzed is shown in Fig 2A and S3 Table. While the majority of the isolates (78%) had a high level of assignment to only one cluster (Q > 0.80; colors in bars in Fig 2 represent scores listed in S3 Table), 7 *T*. *b*. *rhodesiense*, 13 *T*. *b*. *brucei*, and 3 *T*. *evansi* isolates showed uncertain assignment to any one of seven clusters (Q < 0.80, bars with no single color representing more than 80% in Fig 2) to any one of seven clusters (Fig 2A). This uncertain assignment could be due to a variety of factors, ranging from shared common ancestry or recent admixture to limitations of the genetic markers to separate such recently diverged taxa. Cluster “b” (purple) includes only *T*. *b*. *brucei* isolates and corresponds to the “Kiboko B” group [15]. Cluster “a” (orange), “c” (blue), “d” (green), and “f” (grey) include both *T*. *b*. *brucei* and *T*. *b*. *rhodesiense* isolates. Cluster “g” (red) includes isolates from all the three taxa, *T*. *b*. *brucei*, *T*. *b*. *rhodesiense*, and *T*. *evansi*. Cluster “e” (yellow) includes only *T*. *evansi* isolates.

The level of population structure and grouping we observed for *T*. *brucei* is similar to results from previous microsatellite [15,42] and genomic [13,14] analyses, where *T*. *b*. *rhodesiense* isolates were consistently assigned to multiple clusters together with *T*. *b*. *brucei* isolates. This data confirms multiple independent origins of the human disease parasite, *T*. *b*. *rhodesiense*, from different non-human infective *T*. *b*. *brucei* strains and implies that the SRA gene has moved horizontally between strains, which is consistent with earlier studies and experimental evidence that this can occur in the field [6,13,14,15,42,62,63,64,65,66]. As pointed out previously, this finding has important practical implications for disease control and monitoring, as it provides further evidence that *T*. *b*. *brucei* strains can relatively easily transform into *T*. *b*. *rhodesiense* strains and pose a serious risk to human health [13,14].

The STRUCTURE results for *T*. *evansi* isolates are displayed in detail in Fig 2B. Also included are the results of the RoTat 1.2 PCR assay and information on the kDNA minicircle type (based on the literature, where available, or as predicted from our A281del PCR assays; see Tables 1 and S1). Although the majority of *T*. *evansi* isolates assigned to cluster “e” (yellow), there are 6 isolates that assigned with high Q values (Q > 0.80) to different STRUCTURE-defined genetic clusters, and 3 isolates (STIB810, STIB708 and STIB806K) with uncertain assignment (Q < 0.80). Of the isolates with high Q values to non “e” clusters, one isolate (K2479) assigned to cluster “f” (gray), two isolates (K3552 and K3557) to cluster “c” (blue), and two isolates (RoTat1.2 and STIB811) to cluster “g” (red), implying that some *T*. *evansi* isolates are genetically closer to *T*. *brucei* isolates than to each other and supporting the hypothesis of multiple independent origins of *T*. *evansi* isolates from *T*. *brucei*. All 33 isolates with kDNA minicircle type A were assigned to either cluster “e” or “g”, the single confirmed type B (K2479) assigned to cluster “f”, and the two isolates that could not be classified as type A or type B by our assays (K3552 and K3557) assigned to cluster “c” (Fig 2B). This result suggests an association of kDNA minicircle type A with the “e” and “g” clusters, and that the other isolates in our dataset associated with other dominant minicircle types (S1 Table) are from genetically distinct lineages. In contrast, there was no assignment pattern for the isolates that typed as RoTat 1.2 positive or negative based on the PCR assay (Table 1), as the positive isolates assigned to three different clusters (“c”, “g”, and “e”; Fig 2B). The high virulence isolate, K2479 (a kDNA minicircle type B and RoTat 1.2 negative isolate), grouped with the “f” cluster, while the low virulence isolate, K3576 (a RoTat 1.2 positive isolate) assigns to the “e” cluster (Fig 2B). This separation into different clusters suggests independent evolution, but more samples from different genetic backgrounds and virulence degrees are necessary to validate the generality of this observation.

**Fig 3:**
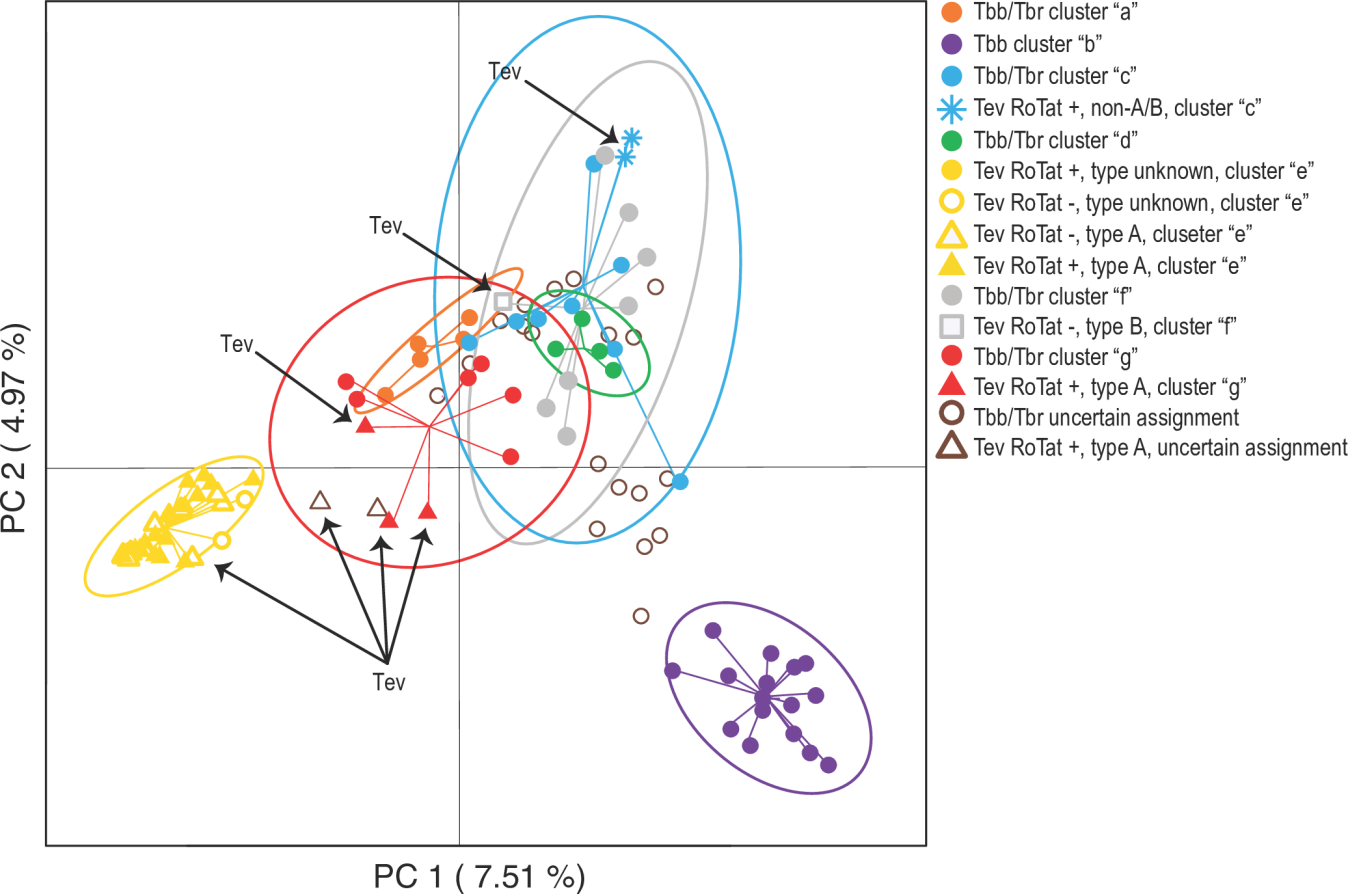
Evaluation of the genetic differentiation between isolates of *Trypanosoma brucei brucei* and *T*. *b*. *rhodesiense* (Tb) and *T*. *evansi* (Tev) genetic clusters using principal components analysis (PCA) of microsatellite data. PCA was performed in R using the package “adegenet” [86]. Points representing individual genotypes are marked by color of their STRUCTURE assignment following the key and connected by a line to the centroid of an ellipse, which circumscribes a region encompassing 95% of the variance observed within each subgroup identified. Black arrows point out the Tev isolates.

The results of the multivariate analyses (PCA, Fig 3) largely confirmed the pattern of genetic structuring suggested by the Bayesian analyses (Fig 2A and 2B) and also provided additional insights on how the different STRUCTURE-based clusters are genetically similar. Individuals from four of five STRUCTURE-defined clusters that include both *T*. *b*. *brucei* and *T*. *b*. *rhodesiense* isolates (clusters “a”, “c”, “d”, “f”, and “g”) grouped close together in the multivariate space defined by the first two PC axes, with isolates from the “a” and “g”, and isolates from the “c”, “d”, and “f” clusters being indistinguishable from one another along the first two components (PC 1 and 2). These close genetic relationships were also implied by the uncertain STRUCTURE cluster assignment of some *T*. *brucei*, which suggests some shared ancestry with all these clusters (Fig 2A, bars with no single dominant color representing more than 80% of the size). On the other hand, the *T*. *b*. *brucei* “Kiboko B” isolates (cluster “b”, Fig 2A) were clearly genetically distinct from the other isolates (purple ellipsoid in Fig 3), as also suggested by the high Q values assignment of these isolates to a single STRUCTURE-based cluster (Fig 2A). The isolates included in STRUCTURE-based cluster “e” (yellow in Fig 2A, exclusively *T*. *evansi* isolates), were also separate from the others. However, they were proximal to cluster “g” isolates and to two *T*. *evansi* isolates with uncertain assignment (Fig 2B), indicating a close evolutionary relationship between the *T*. *evansi* and *T*. *brucei* isolates in these two clusters (Fig 3). As for the STRUCTURE analyses, some *T*. *evansi* isolates were closer to *T*. *brucei* isolates included in different clusters (“f”, “c”, and “g"; Fig 2B). Thus, both Bayesian and multivariate analyses suggest that some *T*. *evansi* isolates share closer evolutionary relationships with different *T*. *brucei* isolates than with each other.

### Genetic diversity and levels of differentiation

To compare diversity and differentiation within and among *T*. *evansi* and *T*. *brucei*, we estimated basic diversity statistics, genetic distance, and F_ST_ among STRUCTURE-based clusters at three levels defined as follows: (i) all of the 84 isolates with Q > 0.80 regardless of taxonomy (S3 Table), (ii) the 46 *T*. *brucei* isolates with Q > 0.80 (S3A Table), and (iii) the 38 *T*. *evansi* isolates with Q > 0.80 (S3B Table). Basic diversity statistics are shown in Table 2. Allelic richness within clusters of all isolates (Table 2A) ranged from 2.10 in cluster "d" to 3.86 in cluster "f", indicating the lowest genetic diversity in cluster “d” that contains both *T*. *b*. *brucei* and *T*. *b*. *rhodesiense*, but not *T*. *evansi* (Fig 2), and the highest genetic diversity in cluster “f” that contains *T*. *b*. *brucei*, *T*. *b*. *rhodesiense*, and *T*. *evansi* (Fig 2). Observed and expected heterozygosity levels and the related inbreeding coefficient (F_IS_) are also reported in Table 2A. Within clusters including all isolates, H_O_ ranged from 0.50 in cluster “g” to 0.66 in cluster “e”, H_E_ ranged from 0.47 in cluster “d” to 0.78 in cluster “g”, and F_IS_ ranged from -0.30 in cluster "e" to 0.34 in cluster "f", spanning a wide range of heterozygosity and conformity to the expectations of Hardy-Weinberg (H-W) equilibrium. This is not surprising given the importance of random mating and sexual reproduction in the maintenance of H-W equilibrium, and the known variation of these life history traits among trypanosome taxa [67,68]. For *T*. *brucei* only isolates (Table 2B), within cluster allelic richness estimates were very similar but slightly lower than the estimates based on all isolates (Table 2A). H_O_ ranged from 0.50 to 0.63, H_E_ ranged from 0.47 to 0.76, and F_IS_ values were mostly positive, ranging from -0.18 to 0.36 (Table 2B). Thus, *T*. *brucei* observed and expected hetrozygosity and F_IS_ values indicate moderate deviation from H-W expectations, and are similar to those reported in a previous study [42], where F_IS_ ranged from -0.16 to 0.43. For *T*. *evansi* only isolates (Table 2C), within cluster allelic richness was intermediate to that found in *T*. *brucei*, indicating genetic diversity similar to that found in *T*. *brucei*. H_O_ ranged from 0.40 to 0.69, H_E_ ranged from 0.36 to 0.72, and F_IS_ values ranged from -0.30 to 0.19. Negative F_IS_ in some clusters in both *T*. *brucei* and *T*. *evansi* could result from clonal, non-sexual reproduction (as expected for the latter) because there is a well understood decrease in expected heterozygosity during clonal reproduction, which lowers F_IS_ [69]. The finding of relatively high allelic richness in all clusters and both positive and negative F_IS_ values in both *T*. *brucei* (Table 2B) and *T*. *evansi* (Table 2C) could be a reflection of different relative levels of sexual and clonal reproduction and recombination among *T*. *brucei* isolates in different clusters, and to the fact that for *T*. *evansi* isolates are strictly clonal.

**Table 2:** Genetic diversity found within each STRUCTURE-based [51] genetic clusters considering (A) all isolates, (B) *T*. *brucei* (Tb) isolates only, and (C) *T*. *evansi* (Tev) isolates only. Sample size within the cluster (N), allelic richness (A_R_) calculated in *FSTAT v1*.*2* [54], and observed heterozygosity (H_O_), expected heterozygosity under Hardy-Weinberg expectations (H_E_), and the inbreeding coefficient (F_IS_) calculated in the R package *HIERFSTAT v0*.*4–10* [55]. Allelic richness could not be calculated in clusters made up of less than 4 individuals (marked n/a).

**A.**	**N**	**A**_**R**_	**H**_**O**_	**H**_**E**_	**F**_**IS**_
**“a” (orange)**	6	2.55	0.58	0.57	-0.02
**“b” (purple)**	16	3.07	0.55	0.61	0.10
**“c” (blue)**	10	3.67	0.63	0.76	0.16
**“d” (green)**	4	2.10	0.55	0.47	-0.20
**“e” (yellow)**	33	2.35	0.66	0.51	-0.30
**“f” (grey)**	8	3.86	0.53	0.78	0.34
**“g” (red)**	9	3.48	0.50	0.71	0.31
**Overall**	**86**	**3.01**	**0.57**	**0.63**	**0.10**
**B.**	**N**	**A**_**R**_	**H**_**O**_	**H**_**E**_	**F**_**IS**_
**Tb “a” (orange)**	6	2.55	0.58	0.56	-0.02
**Tb “b” (purple)**	16	3.07	0.55	0.61	0.10
**Tb “c” (blue)**	8	3.70	0.63	0.76	0.19
**Tb “d” (green)**	4	2.10	0.55	0.47	-0.18
**Tb “f” (grey)**	7	3.77	0.50	0.76	0.36
**Tb “g” (red)**	6	3.22	0.55	0.66	0.22
**Tb overall**	**47**	**3.07**	**0.56**	**0.64**	**0.11**
**C**	**N**	**A**_**R**_	**H**_**O**_	**H**_**E**_	**F**_**IS**_
**Tev “c/f” (blue/grey)**	3	n/a	0.69	0.72	0.06
**Tev “e” (yellow)**	33	2.35	0.66	0.51	-0.30
**Tev “g” (red)**	3	n/a	0.40	0.36	0.19
**Tev overall**	**39**	**2.35**	**0.58**	**0.59**	**-0.09**

To evaluate if levels of genetic differentiation among *T*. *evansi* isolates were different from the ones observed among *T*. *brucei* isolates, we estimated pairwise genetic distances, using Reynolds distances. First, we estimated a distance tree using all the 107 isolates (S4 Fig). This tree clustered the *T*. *evansi* isolates in four different groups, confirming the results of both Bayesian and multivariate analyses (Figs 2 and 3), although bootstrap values among these groups are not high, thus limiting the strength of the inference that can be drawn from this analysis. Next, we estimated within-cluster distances using the STRUCTURE-defined clusters, including only the 84 isolates with Q > 0.8 (S3 Table), as described for the estimates of basic diversity statistics (Table 2). Within-cluster mean distances among all isolates (S4A Table) averaged 0.70 and ranged from 0.57 in cluster “e” to 0.80 in cluster “f”, indicating that the lowest within-cluster distance occurs in the *T*. *evansi* only cluster, and the highest within-cluster distance occurs in a cluster that contains *T*. *b*. *brucei*, *T*. *b*. *rhodesiense* and *T*. *evansi* of type B. Within-cluster mean distances among *T*. *brucei* isolates averaged 0.72 and ranged from 0.61 in cluster “d” to 0.81 in cluster “f” (S4B Table, S5 Fig). Finally, within-cluster mean distances among *T*. *evansi* isolates averaged 0.64 and ranged from 0.57 in cluster “e” to 0.75 in cluster “g” (S4C Table, S5 Fig). The implications of these findings for evolutionary origins of *T*. *evansi* are discussed in detail below.

The analysis of variance (ANOVA) indicated that within-cluster distance was significantly dependent on cluster of assignment (p-value < 0.0001). The results of the Tukey-Kramer HSD test are reported in S5 Table. These tests indicated that *T*. *evansi* cluster “e” and *T*. *brucei* cluster “d” had significantly lower within-cluster distance than any other cluster (S4 Table, S5 Fig), suggesting that the most common *T*. *evansi* lineage (cluster “e”) is of recent origin and is made up of more closely related isolates than those included in most *T*. *brucei* clusters (except cluster “d”). However, since this test could only be carried out for one of the *T*. *evansi* clusters, cluster “e”, because of low number of *T*. *evansi* isolates in the other clusters, the generalitiy of this finding remains uncertain without further sampling of a greater diversity of *T*. *evansi* isolates from non “e” clusters.

To compare among-cluster differentiation in *T*. *evansi* and *T*. *brucei*, we estimated among-cluster F_ST_ using the STRUCTURE-defined clusters and only including the 84 isolates with Q > 0.8 (S3 Table), as described for the estimates of basic diversity statistics (Table 2). F_ST_ estimates are reported in S6 Table. Among-cluster F_ST_ estimates between clusters regardless of taxonomy (S6A Table) ranged from 0.08 between clusters “g” and “e” to 0.31 between clusters “a” and “d” and showed significant differentiation between all clusters (p-value < 0.006), indicating that the lowest genetic differentiation was found between two clusters that contained *T*. *evansi* (“g” and “e”), and that the highest genetic differentiation was found between two clusters (“a” and “d”) made up of entirely *T*. *brucei* isolates. Thus, the most common *T*. *evansi* cluster “e” is less differentated from the *T*. *brucei*-only cluster “a” than both *T*. *brucei*-only clusters “a” and “d” are to one another. Among-cluster F_ST_ estimates in *T*. *brucei* (S6B Table) ranged from 0.10 to 0.31 (S6B Table), and showed significant differentiation between all clusters (p-value < 0.005), indicating high levels of genetic differentiation. Among-cluster F_ST_ in *T*. *evansi* (S6C Table) were similar to those in *T*. *brucei*, ranging from 0.06 to 0.29, and showed significant differentiation (p-value < 0.0001) between *T*. *evansi* in all clusters except the least differentiated clusters “e” and “g”, suggesting *T*. *evansi* cluster “e” and “g” are not significantly differentiated from each other. The low sample size of *T*. *evansi* in cluster “g” remains another possible reason for the non-significant p-value in F_ST_ estimates between “e” and “g”, and again highlight the need for further sampling of a greater diversity of *T*. *evansi* strains from non “e” clusters.

These results indicate that the genetic diversity across all *T*. *evansi* isolates (“overall” in Tables 2C and S4C) represents a large amount of the genetic diversity found across *T*. *brucei* isolates (“overall” in Tables 2B and S4B). However, within clusters including all isolates, the most common *T*. *evansi* cluster, cluster “e”, shows the least amount of genetic differentiation among isolates and the lowest amount of within-cluster genetic diversity compared to other clusters (Tables 2A and S4A), with only the *T*. *brucei* cluster “d” showing similarly low levels (Tables 2A and S4A). The Chi-square test showed that the time of isolation did not account for cluster assignment (Chi^2^ = 20.19, degrees of freedom = 30, p-value = 0.9113).

### Interpretation of evolutionary origins of *T*. *evansi*

Clustering and diversity analysis indicate that *T*. *evansi* strains likely originated from multiple genetic backgrounds (Figs 2 and 3) and that the genetic diversity harbored by the *T*. *evansi* isolates analyzed in this study encompass a large proportion of the total diversity found in the *T*. *brucei* isolates (Tables 2 and S4). The single type B and the two unclassified isolates fall into distinct clusters ("f" and "c", respectively; Fig 2), while type A isolates separate into two clusters ("e" and "g"; Fig 2), that are closely associated in the multivariate analysis (yellow and red; Fig 3). Cluster "e" is made up entirely of *T*. *evansi* isolates (Figs 2 and 3), while cluster "g" includes a mix of *T*. *b*. *brucei*, *T*. *b*. *rhodesiense*, and *T*. *evansi* (Figs 2 and 3). Separation of type A into two closely related clusters suggests that the *T*. *evansi* only cluster "e" has evolved from within cluster "g", and both have evolved from the same *T*. *brucei* ancestor. Nonetheless, these results could also indicate that traits that are common between *T*. *evansi* in clusters "e" and "g" have evolved twice, independently. Evidence for these alternative hypotheses remains inconclusive. Support for a single origin of type A from within cluster "g" comes from the non-significant differentiation (F_ST_) found between the *T*. *evansi* isolates in clusters "e" and "g" (Fst = 0.06, p-value = 0.105; S6C Table), which indicates high similarity between these clusters. Furthermore, certain *T*. *evansi* isolates from China (STIB810, STIB811, and STIB806K) that were isolated within 3 years from each other and presumably are closely related [10,17,36] can be found in both clusters "e" and "g": STIB810 assigns to cluster "e", STIB811 assigns to cluster "g", and STIB806K assigns about equally to both "e" and "g" (Fig 2), suggesting the “e” and “g” clusters are not the result of distinct geographic origins or outbreaks. Thus, distinct clustering of type B in cluster "f", distinct clustering of unclassified isolates in cluster "c", and nested clustering of type A isolates in the two closely related clusters "e" and "g" suggests independent origins of each *T*. *evansi* kDNA type from a diverse *T*. *brucei* background.

### Comparisons with previous studies

The results from our screen of 15 microsatellite loci largely aligns with previous phylogenetic and population genetic analyses, which indicated that *T*. *evansi* strains are nested phylogenetically within the more genetically diverse *T*. *brucei* [1,10,11,13,17,70], likely originated from different *T*. *b*. *brucei* strains [10,70], and are highly variable [35,70]. Some studies [70–72] found that the *T*. *evansi* strains sampled clustered closely with one another and separately from *T*. *b*. *brucei* and *T*. *b*. *rhodesiense* strains. We suggest that this pattern of genetic similarity can be an artifact resulting from the limited number and type of isolates included in these studies. This is especially true for the *T*. *evansi* isolates that only included the common kDNA type A lineage (i.e. kDNA minicircle type A configuration and RoTat 1.2 positive). Indeed, other studies that have included both type A and type B *T*. *evansi* isolates have found similar results to what we have found, using a larger geographic and taxonomic diversity of isolates [10,17,35,73]. Interestingly, our findings are also consistent with previous comparative genomic analysis [10] and with classical parasitological characterization, which indicates high similarity between *T*. *evansi* and *T*. *b*. *brucei* except for variable patterns of loss of part or all of their kDNA [1,12,16,74].

## Conclusions and future directions

This work shows that *T*. *evansi* strains from Eastern Africa, the main region where both *T*. *evansi* and *T*. *b*. *brucei* strains co-occur, likely originated from multiple *T*. *b*. *brucei* strains and harbor a high degree of circulating genetic variation. This result is surprising because of the phenotypic similarities between all *T*. *evansi* strains, such as ability to sustained mechanical transmission outside the tsetse belt, variable loss of functional kDNA, and the common disease symptoms they cause in a variety of animals. Multiple origins of *T*. *evansi* phenotypes implies that complex traits such as ability for mechanical transmission have evolved multiple times, and that there is plenty of standing genetic diversity to provide opportunity for selection to generate novel strains. Further research is needed to understand the mechanism of this evolutionary transition.

Our results provide further support for the idea that the taxonomic rank of *T*. *evansi* is not valid from an evolutionary standpoint [10,12,17,75]. However, even the subspecies designation suggested by some authors is not taxonomically correct, since this rank should, by definition, be used to identify groups of populations within a species that are geographically and genetically differentiated. We propose that the taxonomy of the groups within the genus *Trypanosoma*, including *T*. *b*. *rhodesiense*, *T*. *evansi* and *T*. *equiperdum*, requires a fundamental revision that, as proposed by Gibson [67], should ‘bring together considerations of utility, genetic difference and adaptation’.

These findings mirror what is known about the multiple evolutionary origins of *T*. *b*. *rhodesiense* from different strains of the animal parasite *T*. *brucei brucei*, and thus highlight the trypanosome’s ability to evolve novel and complex traits to expand their host repertoire. This has important epidemiological implications, as *T*. *b*. *brucei* strains from different genetic backgrounds apparently can become either parasites of a lethal human disease (i.e. *T*. *b*. *rhodesiense*) [76,77]or become able to be transmitted by a variety of hematophagous insects besides the tsetse fly (i.e. *T*. *evansi*) [10,68,76]. To date, there have been only few reported cases of *T*. *evansi* infecting humans [78] a well-documented case from India was thought to be non-transmissible to other humans with fully functioning immune systems [79]. Thus, risk of human infective *T*. *evansi* remains theoretical, but deserves consideration since this would allow human sleeping sickness to escape sub-Saharan Africa and take advantage of hosts worldwide. In order for the human disease to escape sub-Saharan Africa, both mechanical transmission and evasion of the human immune system would be needed in a single strain. The fact that trypanosomes have been able to acquire both traits repeatedly makes the acquisition of both features in one strain a dangerous possibility. However, this possibility remains remote for several reasons. First, mechanical transmission in human infective strains would require much higher levels of parasitemia than observed in infections caused by *T*. *b*. *gambiense* [7,80], the subspecies responsible for the vast majority of cases of human African trypanosomiasis. Second, the acquisition of the SRA gene requires sexual recombination in the tsetse fly, which does not occur in *T*. *evansi* once it has become dyskinetoplastic. Nonetheless, if this were to happen, the spread of sleeping sickness outside of sub-Saharan Africa would have dramatic consequences because diagnosis is complicated, pharmacological therapy is inadequate [81–83], and vaccines are non-existent.

Future work should therefore focus on understanding the origin and dynamics of the *T*. *evansi* spatial expansion from Africa to multiple continents, as well as on the functional and molecular basis of the ability to by-pass tsetse flies for their transmission. Screening for genetic polymorphism in additional *T*. *evansi* isolates from across the world will help us understand the origin and timing of the *T*. *evansi* expansion, evaluate if only a few genetically similar strains were responsible for the spread, and identify the *T*. *brucei* genetic background most likely to give rise to *T*. *evansi* strains. Adding genome-wide data will provide higher resolution of the phylogenetic relationships among these strains and insights on the genetic, functional and molecular basis of novel complex traits such as “mechanical transmission”.

## Supporting information

S1 Fig Diagnostic PCR for the GCT/Ala281 deletion in F1FO-ATP synthase subunit γ in *T*. *evansi* type A. Shown are nucleotides 1–859 (GCT deletion) and 1–863 (‘wild type’), respectively, of gene TevSTIB805.10.220 / Tb427.10.180 (systematic TriTrypDB.org IDs). Primer combination F1/R1 will give a 855-bp amplicon if the deletion is present. Primer combination F1/R2 will give a 863-bp amplicon for most if not all isolates from the group of 5 closely related named taxa includes *T*. *evansi* (also known as subgenus *Trypanozoon*).
(TIF)

S2 Fig STRUCTURE v2.3.4 [51] plot of delta K for K values of 2 to 9 based on 20 runs each performed with a burn-in of 5,000 and a total of 250,000 iterations. Although K = 2 had the highest delta K and thus explained the highest hierarchical level in the data, a K value of 7 was the next hierarchical level with a peak in delta K, and was able to distinguish structure within *Trypanosoma brucei brucei* and *T*. *b*. *rhodesiense*. See S3 Fig for display of K = 2.
(TIF)

S3 Fig STRUCTURE v2.3.4 [51] plot of individual assignments with K values of 2 through 7. Each vertical bar represents a strain’s probability of assignment to one of K genetic clusters, with *T*. *brucei* (Tb) strains on the left (light gray horizontal bar) and *T*. *evansi* (Tev) strains on the right (dark gray horizontal bar). Individuals with 100% probability of assignment to one cluster are represented by bars of only one color, individuals with multiple assignment to different genetic cluster are represented by bars with multiple colors.
(TIF)

S4 Fig Distance tree based on 15 microsatellite markers and Reynolds et al (1983) distances using the UPGMA method implemented in the R package, “PopPR” *v2*.*3* [54, 55]. Support values are shown on nodes only for values above 50% and are based on 1000 bootstrap replicates. Terminal tips identify the strains (Table 1 and S1 Table) and are color coded according to the upper left legend with respect to their STRUCTURE-defined cluster assignment and the results of the diagnostic PCR assays (Table 1). The major *T*. *evansi* cluster is shown with a black vertical bar, and the other *T*. *evansi* strains are marked with black arrows.
(TIF)

S5 Fig Summary of pairwise Reynolds (1983) genetic distances computed in the R package. “PopPR” *v2*.*3*.*0* [54, 55] between strains belonging to the same or different STRUCTURE-defined clusters as outlier box-plots color coded according to legend to the left. Boxes and whiskers on each box-plot represent the minimum, 1st quartile, 3rd quartile, and maximum distances. Panel **(A)** displays distances between a *T*. *brucei* strain and a *T*. *evansi* strain, panel **(B)** displays distances between two *T*. *brucei* strains, and panel **(C)** displays distances between two *T*. *evansi* strains. Each symbol (¥, §, †, •, and *) represents a group of statistically distinct within-cluster distance based on the analysis of variance (ANOVA, p-value < 0.0001), and the Tukey-Kramer HSD test performed in JMP v11.2 (SAS Institute Inc., Cary, NC, USA, 1989–2012). Boxplots that are not connected with the same symbol contain significantly different levels of among-cluster genetic distances. For example, ¥ joins clusters “d” (green) and “e” (yellow), indicating significantly lower within-cluster distance in these two clusters than any other cluster. See S5 Table for details of the Tukey-Kramer HSD test.
(TIF)

S1 Table Sample details of strains from previous studies showing sample ID, publication, taxon, kDNA, host of isolation, locality of origin and year of isolation, n/a indicates no history found on the year of isolation. (DOCX)

S2 Table PCR primers used in microsatellite marker amplification, with general information about the motif, size range in bp (size), chromosome location (location), and source of the protocol used. (DOCX)

S3 Table Assignment scores from STRUCTURE v2.3.4 [51] clustering analysis with K = 7 showing sample ID, taxon, genetic cluster “a-g” (Fig 2) if probability of assignment (Q) above or equal to 0.8, or "uncertain" if Q < 0.8 for each strain of **(A)** *Trypanosoma brucei brucei* (Tbb) or *T*. *b*. *rhodesiense* (Tbr), and **(B)** *T*. *evansi* (Tev).
(DOCX)

S4 Table Within-cluster distance using STRUCTURE-based [51] genetic clusters including strains with Q values > 0.80 (S3 Table) for **(A)** all strains regardless of taxonomy, **(B)** *T*. *brucei* (Tb) strains, and **(C)** *T*. *evansi* (Tev) strains. Number of pairwise between-strain comparisons (N pairs), mean Reynolds (1983) [56] distance (mean distance) estimated in the R package “PopPR” *v2*.*3*.*0* [57, 58], standard deviation (SD), minimum distance (min), and maximum distance (max).
(DOCX)

S5 Table Summary of differences in within-cluster Reynolds [56] distance of STRUCTURE-defined clusters based on analysis of variance (ANOVA, p-value < 0.0001), and the Tukey-Kramer HSD test performed in JMP v11.2 (SAS Institute Inc., Cary, NC, USA, 1989–2012), using only the 86 strains with Q values >0.80 (S3 Table): **(A)** Ordered difference report between clusters showing the clusters compared (cluster 1 and cluster 2), the difference in mean Reynolds distance (Dif), the standard error of the difference (Std Err Dif), the lower confidence level (CL), the upper confidence level (CL), and the p-value of the pairwise comparison. **(B)** The connecting symbols report that summarizes the Tukey-Kramer HSD tests, where each symbol group (¥, §, †, •, *) contain significantly different within-cluster pairwise genetic distances (¥ joins clusters “d” and “e”, § joins clusters “a” and “d”; † joins clusters “a”, “b”, and “c”; • joins clusters “a”, “b”, “c”, and “g”; and * joins clusters “c”, “f”, and “g”).
(DOCX)

S6 Table Among-cluster genetic differentiation (F_ST_) among each STRUCTURE-defined [51] genetic cluster, using only strains with Q values >0.80 (S3 Table): **(A)** all strains, **(B)** *T*. *brucei* (Tb) strains only, and **(C)** *T*. *evansi* (Tev) strains only. Pairwise F_ST_ (below diagonal) was calculated in ARLEQUIN *v*.*3*.*2* [59] with Wright’s statistics [60], following the variance method developed by Weir and Cockerham (1984) [61] using 10,000 permutations to obtain exact p-values (above diagonal), with the only non-significant F_ST_ found (between *T*. *evansi* cluster “e” and “g”) in bold.
(DOCX)
