# Supplementary figures and images for: Multiple evolutionary origins of Trypanosoma evansi in Kenya
Source: PLoS Negl Trop Dis. 2017 Sep 7;11(9):e0005895. doi: 10.1371/journal.pntd.0005895 (PMC5605091; doi:10.1371/journal.pntd.0005895)

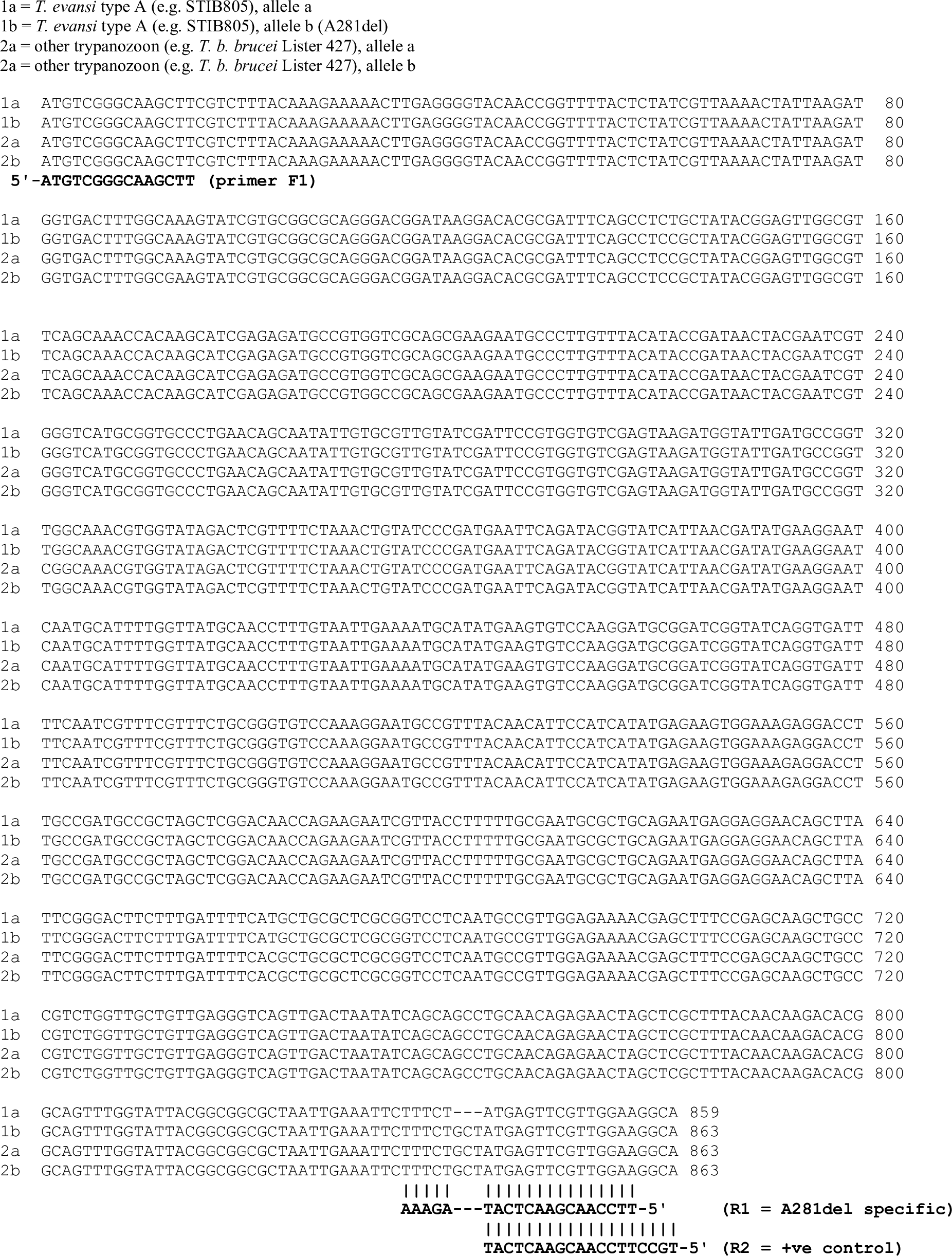

Supplement: S1 Fig — Shown are nucleotides 1–859 (GCT deletion) and 1–863 (‘wild type’), respectively, of gene TevSTIB805.10.220 / Tb427.10.180 (systematic TriTrypDB.org IDs). Primer combination F1/R1 will give a 855-bp amplicon if the deletion is present. Primer combination F1/R2 will give a 863-bp amplicon for most if not all isolates from the group of 5 closely related named taxa includes T. evansi (also known as subgenus Trypanozoon). (TIF) [file pntd.0005895.s001.tif]

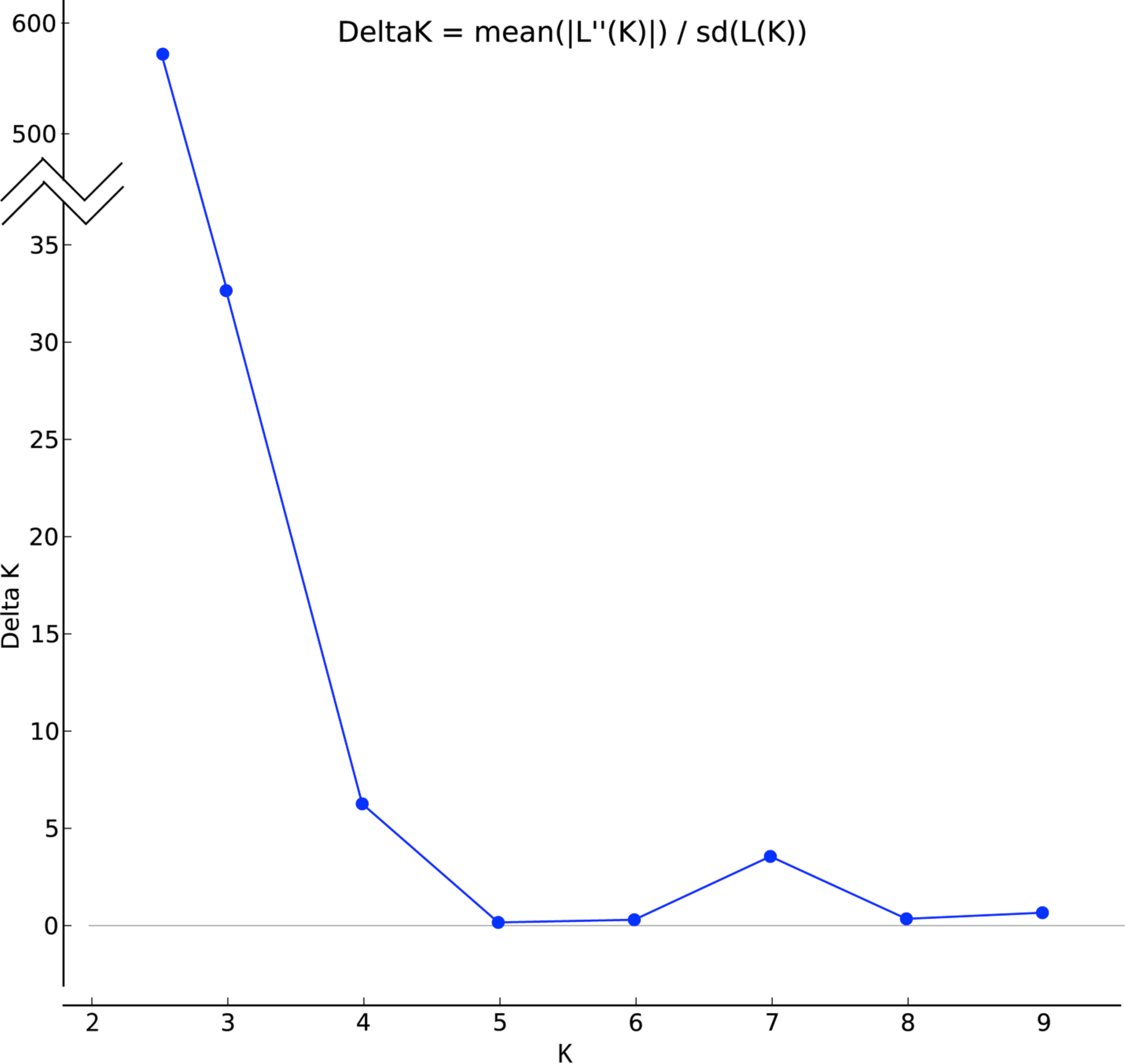

Supplement: S2 Fig — Although K = 2 had the highest delta K and thus explained the highest hierarchical level in the data, a K value of 7 was the next hierarchical level with a peak in delta K, and was able to distinguish structure within Trypanosoma brucei brucei and T. b. rhodesiense. See S3 Fig for display of K = 2. (TIF) [file pntd.0005895.s002.tif]

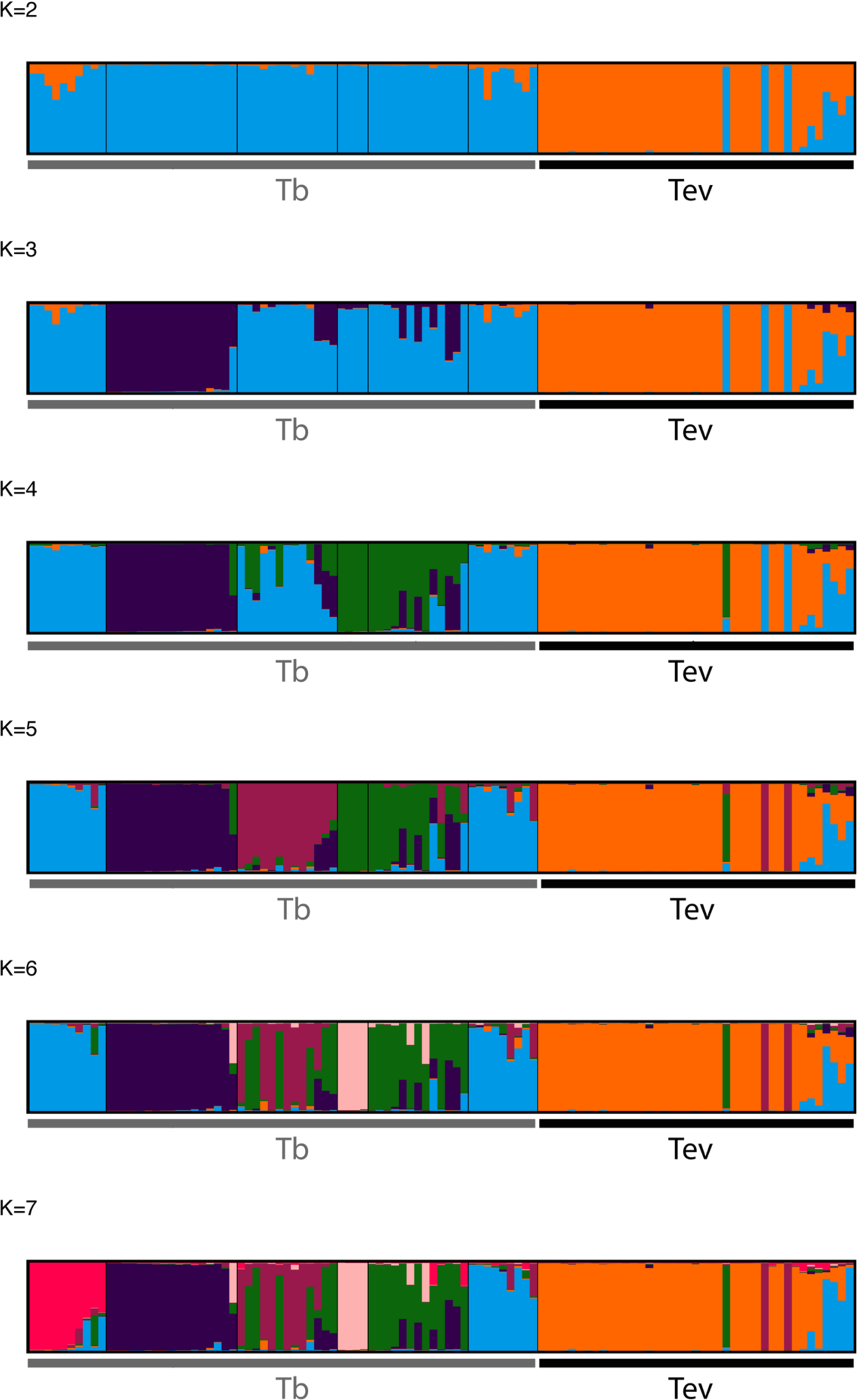

Supplement: S3 Fig — Each vertical bar represents a strain’s probability of assignment to one of K genetic clusters, with T. brucei (Tb) strains on the left (light gray horizontal bar) and T. evansi (Tev) strains on the right (dark gray horizontal bar). Individuals with 100% probability of assignment to one cluster are represented by bars of only one color, individuals with multiple assignment to different genetic cluster are represented by bars with multiple colors. (TIF) [file pntd.0005895.s003.tif]

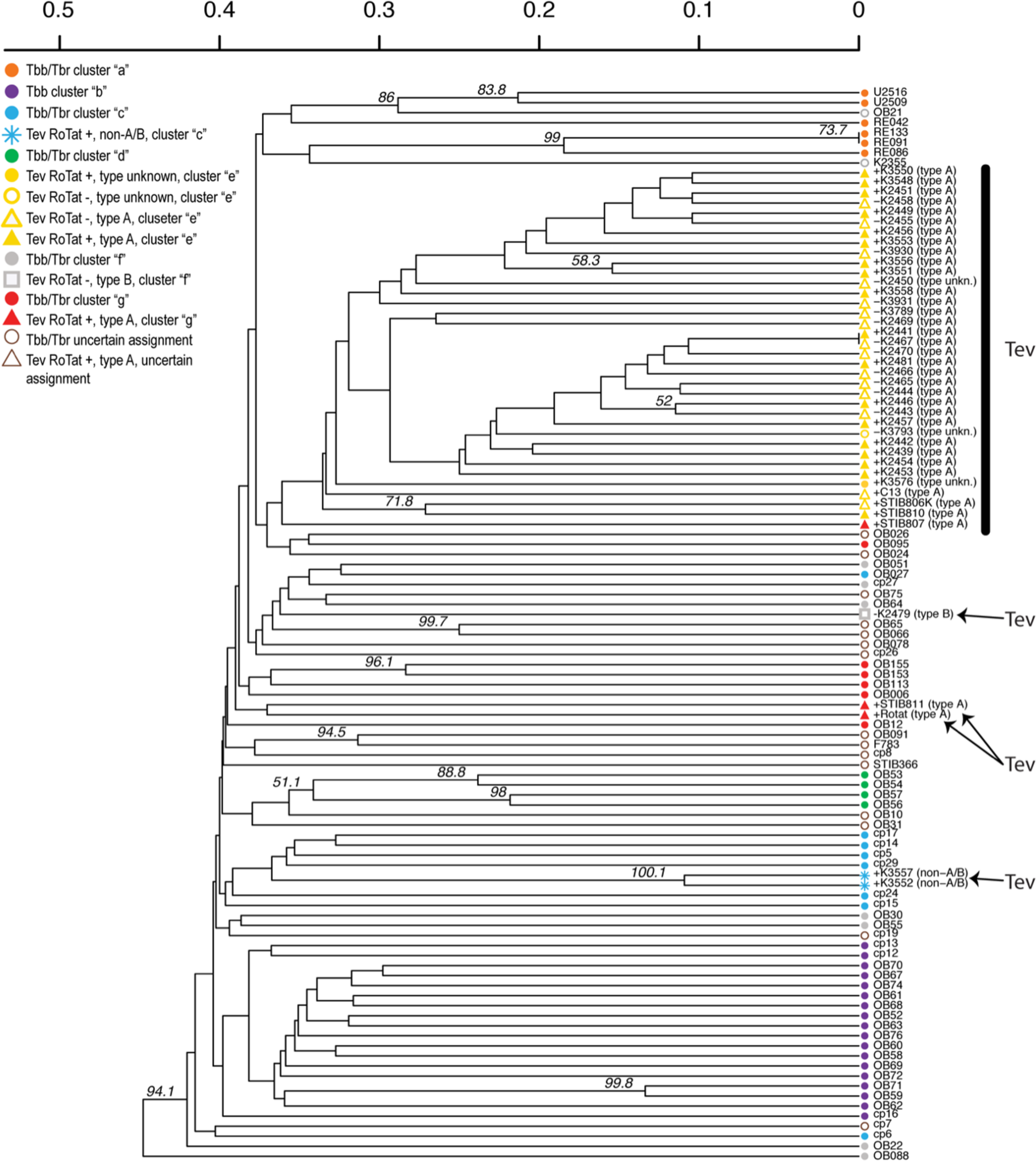

Supplement: S4 Fig — Support values are shown on nodes only for values above 50% and are based on 1000 bootstrap replicates. Terminal tips identify the strains (Table 1 and S1 Table) and are color coded according to the upper left legend with respect to their STRUCTURE-defined cluster assignment and the results of the diagnostic PCR assays (Table 1). The major T. evansi cluster is shown with a black vertical bar, and the other T. evansi strains are marked with black arrows. (TIF) [file pntd.0005895.s004.tif]

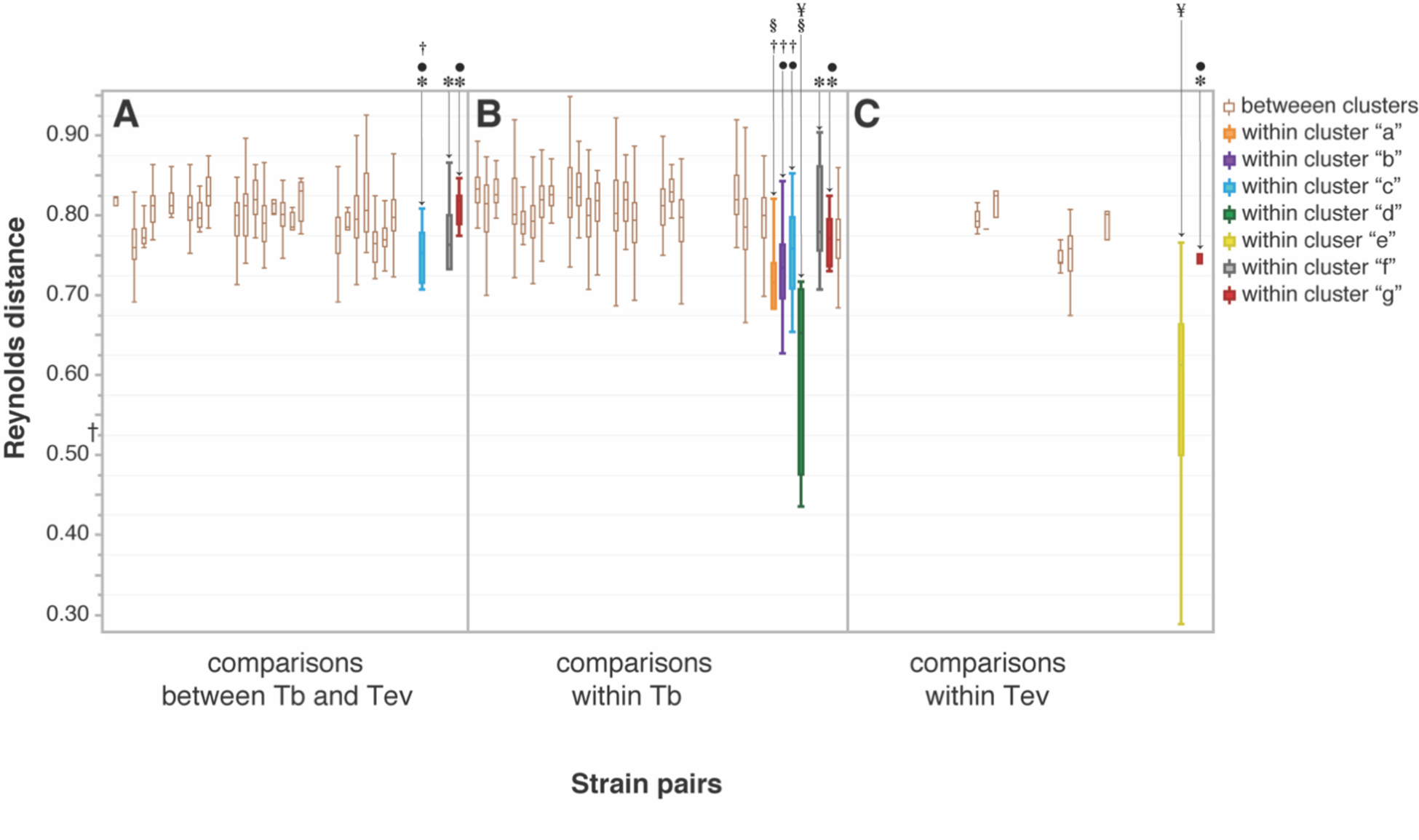

Supplement: S5 Fig — “PopPR” v2.3.0 [54, 55] between strains belonging to the same or different STRUCTURE-defined clusters as outlier box-plots color coded according to legend to the left. Boxes and whiskers on each box-plot represent the minimum, 1st quartile, 3rd quartile, and maximum distances. Panel (A) displays distances between a T. brucei strain and a T. evansi strain, panel (B) displays distances between two T. brucei strains, and panel (C) displays distances between two T. evansi strains. Each symbol (¥, §, †, •, and *) represents a group of statistically distinct within-cluster distance based on the analysis of variance (ANOVA, p-value < 0.0001), and the Tukey-Kramer HSD test performed in JMP v11.2 (SAS Institute Inc., Cary, NC, USA, 1989–2012). Boxplots that are not connected with the same symbol contain significantly different levels of among-cluster genetic distances. For example, ¥ joins clusters “d” (green) and “e” (yellow), indicating significantly lower within-cluster distance in these two clusters than any other cluster. See S5 Table for details of the Tukey-Kramer HSD test. (TIF) [file pntd.0005895.s005.tif]
