# Supplementary material for: Multiple evolutionary origins of Trypanosoma evansi in Kenya
Source: PLoS Negl Trop Dis. 2017 Sep 7;11(9):e0005895. doi: 10.1371/journal.pntd.0005895 (PMC5605091; doi:10.1371/journal.pntd.0005895)
Supplement: S1 Table — (DOCX) [file pntd.0005895.s006.docx]

**S1 Table.** Sample details of strains from previous studies showing sample ID, publication, taxon, kDNA, host of isolation, locality of origin and year of isolation, n/a indicates no history found on the year of isolation.

| **Sample ID** | **Publication** | **Taxon** | **kDNA type** | **Host** | **Locality of origin** | **Year of isolation** |
| --- | --- | --- | --- | --- | --- | --- |
| RoTat1.2 (OB106) | [10,15, 23, 35] | Tev | A | Water buffalo | Indonesia | 1982 |
| STIB708 (KETRI2489, OB35) | [10,15] | Tev | A | Camel | Kenya | n/a |
| STIB806K (OB2) | [10, 36] | Tev | A | Buffalo | China | 1983 |
| STIB811 (OB42) | [10, 36] | Tev | A | Buffalo | China | 1982 |
| RE091 | [42] | Tbb |  | Porcine | Uganda | 2001 |
| RE133 | [42] | Tbb |  | Porcine | Uganda | 2001 |
| RE086 | [42] | Tbb |  | Porcine | Uganda | 2001 |
| UTRO2509 | [42] | Tbb |  | Human | Uganda | n/a |
| UTRO2516 | [42] | Tbb |  | Human | Uganda | 1979 |
| RE042 | [42] | Tbr |  | Human | Uganda | 2010 |
| OB21 | [15] | Tbr |  | *Glossina pallidipes* | Uganda | 1960 |
| F783 | [15] | Tbb |  | n/a | Uganda | 1969 |
| K2355 | [77] | Tbr |  | Human | Uganda | 1977 |
| OB091 | [15] | Tbb |  | n/a | Uganda | 1969 |
| OB59 | [15] | Tbb |  | Lion | Tanzania | 1971 |
| OB71 | [15] | Tbb |  | Lion | Tanzania | 1971 |
| OB67 | [15] | Tbb |  | Lion | Tanzania | 1971 |
| OB63 | [15] | Tbb |  | Lion | Tanzania | 1971 |
| OB68 | [15] | Tbb |  | Lion | Tanzania | 1971 |
| OB61 | [15] | Tbb |  | Lion | Tanzania | 1971 |
| OB52 | [15] | Tbb |  | n/a | Kenya | n/a |
| OB74 | [15] | Tbb |  | Lion | Tanzania | 1971 |
| OB69 | [15] | Tbb |  | Lion | Tanzania | 1971 |
| cp12 | [15] | Tbb |  | Cow | Kenya | 1973 |
| cp16 | [15] | Tbb |  | *Glossina pallidipes* | Kenya | 1969 |
| OB70 | [15] | Tbb |  | Lion | Tanzania | 1971 |
| OB76 | [15] | Tbb |  | Lion | Tanzania | 1971 |
| OB62 | [15] | Tbb |  | Lion | Tanzania | 1971 |
| OB72 | [15] | Tbb |  | Lion | Tanzania | 1971 |
| cp13 | [15] | Tbb |  | Cow | Kenya | 1973 |
| cp6 | [15] | Tbb |  | Lion | Zambia | 1974 |
| cp17 | [15] | Tbb |  | Cow | Kenya | 1980 |
| cp24 | [15] | Tbb |  | Giraffe | Zambia | 1982 |
| cp29 | [15] | Tbb |  | *Glossina morsitans* | Zambia | 1983 |
| cp15 | [15] | Tbb |  | *Glossina pallidipes* | Kenya | 1969 |
| cp5 | [15] | Tbb |  | *Glossina pallidipes* | Kenya | 1983 |
| cp14 | [15] | Tbb |  | *Glossina pallidipes* | Kenya | 1980 |
| OB58 | [15] | Tbb |  | Kongoni | Uganda | 1970 |
| OB56 | [15] | Tbb |  | Hyena | Tanzania | 1971 |
| OB54 | [15] | Tbr |  | Lion | Tanzania | 1971 |
| OB53 | [15] | Tbr |  | n/a | Tanzania | 1971 |
| OB57 | [15] | Tbr |  | Hyena | Tanzania | 1971 |
| OB31 | [15] | Tbb |  | Bovine | Somalia | 1985 |
| OB10 | [15] | Tbb |  | Kongoni | Tanzania | 1971 |
| cp19 | [15] | Tbb |  | Sheep | Kenya | 1981 |
| OB027 | [15] | Tbr |  | Human | Uganda | 1961 |
| cp26 | [15] | Tbb |  | *Glossina pallidipes* | Zambia | 1983 |
| cp7 | [15] | Tbb |  | Hyena | Zambia | 1973 |
| cp27 | [15] | Tbb |  | *Glossina morsitans* | Zambia | 1983 |
| OB30 | [15] | Tbb |  | *Glossina fuscipes* | Uganda | 1971 |
| OB088 | [15] | Tbb |  | *Glossina pallidipes* | Kenya | 1969 |
| OB22 | [15] | Tbr |  | Human | Tanzania | 1982 |
| OB051 | [15] | Tbb |  | Hippo | Uganda | 1961 |
| OB64 | [15] | Tbb |  | Kongoni | Tanzania | 1970 |
| OB55 | [15] | Tbb |  | Hyena | Tanzania | 1971 |
| OB078 | [15] | Tbr |  | Human | Kenya | 1961 |
| OB066 | [15] | Tbr |  | Kongoni | Tanzania | 1970 |
| STIB366 | [15] | Tbb |  | n/a | Uganda | 1966 |
| OB75 | [15] | Tbb |  | Hyena | Tanzania | 1971 |
| OB65 | [15] | Tbr |  | Kongoni | Tanzania | 1970 |
| OB60 | [15] | Tbb |  | Lion | Tanzania | 1971 |
| OB153 | [15] | Tbb |  | Porcine | Cameroon | 1999 |
| OB155 | [15] | Tbb |  | Porcine | Cameroon | 1999 |
| OB095 | [15] | Tbr |  | n/a | Ethiopia | 1967 |
| OB006 | [15] | Tbr |  | Human | Uganda | 1961 |
| OB113 | [15] | Tbb |  | Ox | Burkina Faso | 1989 |
| cp8 | [15] | Tbb |  | Cow | Uganda | 1990 |
| OB026 | [15] | Tbr |  | Human | Ethiopia | 1970 |
| OB12 | [15] | Tbb |  | Kongoni | Tanzania | 1971 |
| OB024 | [15] | Tbr |  | Human | Kenya | 1980 |
