# Supplementary material for: Multiple evolutionary origins of Trypanosoma evansi in Kenya
Source: PLoS Negl Trop Dis. 2017 Sep 7;11(9):e0005895. doi: 10.1371/journal.pntd.0005895 (PMC5605091; doi:10.1371/journal.pntd.0005895)
Supplement: S2 Table — (DOCX) [file pntd.0005895.s007.docx]

**S2 Table.** PCR primers used in microsatellite marker amplification, with general information about the motif, size range in bp (size), chromosome location (location), and source of the protocol used.

| **Locus** | **Forward Primer** | **Reverse Primer** | **Motif** | **Size** | **Location** | **Source** |
| --- | --- | --- | --- | --- | --- | --- |
| TB8/11 | [FAM]-TGTAGCAGTGGTACGCAC | CACCCAACGCATGTAAGC | AT | 97–127 | 8 | [50] |
| TB2/19 | [HEX]-CTGGTGCGTGTAACTGTG | GAAGTGAGGACATGCACG | AT | 84–104 | 2 | [50] |
| TB11/13 | [FAM]-CAAGAACTCTGCATTGAGC | ATCTGTTGGCGATGGTGA | AT | 125-161 | 11 | [50] |
| TB6/7 | [HEX]-AAGCTGACAGGTGGTTGA | GAACATGCGTGCGTGTG | AT | 104-136 | 6 | [50] |
| TB1/8 | [FAM]-AGGTTTAGTGCATGTCGGA | CCTGTTGTACGGAGGTCA | CA | 97-117 | 1 | [50] |
| TB5/2 | [HEX]-CAACCGAAAGTAAGGGGAAC | TCTCGCCTTCTTTGCCC | AT | 83-107 | 5 | [50] |
| TB10/5 | [FAM]-AAAGGCGATATGTTATTATTGA | ATTGGGTATACTGTCCCTCA | TA | 79-115 | 10 | [50] |
| TB9/6 | [HEX]-TGATTCATTGGTTAAGACAGG | AATGATAACTGCGGATTACAC | AC | 124-158 | 9 | [50] |
| Tryp52 | [ALEXA 532]-GCATCATTGACGTCGACCC | TAACAACCACTGGGACCGC | GT | 201-231 | 11 | [49] |
| Tryp54 | [ROX]-AGTCGGCGTGATGGTACTC | TTCAGCCCACAAACAACCG | AAAT | 144-176 | 10 | [49] |
| Tryp55 | [FAM]-AATTCAACCCCAACAGCCC | CTCGTTCAATGACTTGCCCC | GT | 208-246 | 5 | [49] |
| Tryp59 | [ALEXA 532]-GAGGCAATCGCAGTGTGTG | CGCACGTTTCACCATCCTC | GT | 209-225 | 9 | [49] |
| Tryp62 | [ROX]-AAGGCGACCAACTTCAACC | GTTGTCATCGGCTTGCTCC | AC | 153-177 | 11 | [49] |
| Tryp65 | [ALEXA 546]-GGAGGTAAACTTGATTCGGGTG | ACGACAACAGCGACAAAGC | ATT | 207-234 | 9 | [49] |
| Tryp67 | [FAM]-GTTGCTGAGGTGCAACTGG | GTCGTCAGGCACCAAAACG | GTT | 151-178 | 7 | [49] |
