# Supplementary material for: Multiple evolutionary origins of Trypanosoma evansi in Kenya
Source: PLoS Negl Trop Dis. 2017 Sep 7;11(9):e0005895. doi: 10.1371/journal.pntd.0005895 (PMC5605091; doi:10.1371/journal.pntd.0005895)
Supplement: S3 Table — Assignment scores from STRUCTURE v2.3.4 [51] clustering analysis with K = 7 showing sample ID, taxon, genetic cluster “a-g” (Fig 2) if probability of assignment (Q) above or equal to 0.8, or "uncertain" if Q < 0.8 for each strain of (A) Trypanosoma brucei brucei (Tbb) or T. b. rhodesiense (Tbr), and (B) T. evansi (Tev). (DOCX) [file pntd.0005895.s008.docx]

**S3 Table.** Assignment scores from STRUCTURE v2.3.4 [51] clustering analysis with K=7 showing sample ID, taxon, genetic cluster “a-g” (Fig. 2) if probability of assignment (Q) above or equal to 0.8, or "uncertain" if Q < 0.8 for each strain of **(A)** *Trypanosoma brucei brucei* (Tbb) or *T. b. rhodesiense* (Tbr), and **(B)** *T. evansi* (Tev).

| **A.** |  |  |  |  |  |  |  |  |  |
| --- | --- | --- | --- | --- | --- | --- | --- | --- | --- |
| **Sample ID** | **Taxon** | **Genetic cluster** | **“a”** | **“b”** | **“c”** | **“d”** | **“e”** | **“f”** | **“g”** |
| RE091 | Tbb | "a" (orange) | 0.99 | 0.00 | 0.00 | 0.00 | 0.00 | 0.00 | 0.00 |
| RE133 | Tbb | "a" (orange) | 0.99 | 0.00 | 0.00 | 0.00 | 0.00 | 0.00 | 0.00 |
| RE086 | Tbb | "a" (orange) | 0.99 | 0.00 | 0.00 | 0.00 | 0.00 | 0.00 | 0.00 |
| UTRO2509 | Tbb | "a" (orange) | 0.98 | 0.00 | 0.00 | 0.00 | 0.01 | 0.00 | 0.00 |
| UTRO2516 | Tbb | "a" (orange) | 0.98 | 0.00 | 0.01 | 0.00 | 0.00 | 0.00 | 0.01 |
| RE042 | Tbr | "a" (orange) | 0.93 | 0.00 | 0.02 | 0.00 | 0.02 | 0.01 | 0.02 |
| OB021 | Tbr | uncertain | 0.75 | 0.00 | 0.18 | 0.00 | 0.02 | 0.01 | 0.05 |
| F783 | Tbb | uncertain | 0.63 | 0.01 | 0.01 | 0.00 | 0.00 | 0.01 | 0.34 |
| K2355 | Tbb | uncertain | 0.51 | 0.00 | 0.08 | 0.00 | 0.01 | 0.35 | 0.04 |
| OB091 | Tbb | uncertain | 0.56 | 0.01 | 0.00 | 0.00 | 0.00 | 0.03 | 0.39 |
| OB59 | Tbb | "b" (purple) | 0.00 | 0.99 | 0.00 | 0.00 | 0.00 | 0.00 | 0.00 |
| OB71 | Tbb | "b" (purple) | 0.00 | 0.99 | 0.00 | 0.00 | 0.00 | 0.00 | 0.00 |
| OB67 | Tbb | "b" (purple) | 0.00 | 0.99 | 0.00 | 0.00 | 0.00 | 0.00 | 0.00 |
| OB63 | Tbb | "b" (purple) | 0.00 | 0.99 | 0.00 | 0.00 | 0.00 | 0.00 | 0.00 |
| OB68 | Tbb | "b" (purple) | 0.00 | 0.98 | 0.00 | 0.00 | 0.00 | 0.00 | 0.00 |
| OB61 | Tbb | "b" (purple) | 0.00 | 0.98 | 0.00 | 0.00 | 0.00 | 0.01 | 0.00 |
| OB52 | Tbb | "b" (purple) | 0.00 | 0.97 | 0.01 | 0.00 | 0.00 | 0.00 | 0.00 |
| OB74 | Tbb | "b" (purple) | 0.01 | 0.98 | 0.00 | 0.01 | 0.00 | 0.00 | 0.00 |
| OB69 | Tbb | "b" (purple) | 0.00 | 0.98 | 0.00 | 0.00 | 0.00 | 0.01 | 0.00 |
| cp12 | Tbb | "b" (purple) | 0.01 | 0.97 | 0.00 | 0.00 | 0.00 | 0.00 | 0.00 |
| cp16 | Tbb | "b" (purple) | 0.01 | 0.97 | 0.01 | 0.00 | 0.00 | 0.01 | 0.00 |
| OB70 | Tbb | "b" (purple) | 0.02 | 0.96 | 0.00 | 0.00 | 0.00 | 0.00 | 0.01 |
| OB76 | Tbb | "b" (purple) | 0.01 | 0.97 | 0.02 | 0.00 | 0.00 | 0.00 | 0.00 |
| OB62 | Tbb | "b" (purple) | 0.00 | 0.94 | 0.00 | 0.01 | 0.02 | 0.01 | 0.01 |
| OB72 | Tbb | "b" (purple) | 0.00 | 0.89 | 0.00 | 0.00 | 0.01 | 0.00 | 0.10 |
| cp13 | Tbb | "b" (purple) | 0.00 | 0.92 | 0.05 | 0.00 | 0.00 | 0.02 | 0.01 |
| cp6 | Tbb | uncertain | 0.01 | 0.36 | 0.53 | 0.00 | 0.00 | 0.09 | 0.01 |
| cp17 | Tbb | "c" (blue) | 0.03 | 0.00 | 0.85 | 0.05 | 0.02 | 0.03 | 0.02 |
| cp24 | Tbb | "c" (blue) | 0.00 | 0.00 | 0.86 | 0.00 | 0.11 | 0.02 | 0.01 |
| cp29 | Tbb | "c" (blue) | 0.07 | 0.00 | 0.87 | 0.01 | 0.00 | 0.04 | 0.01 |
| cp15 | Tbb | "c" (blue) | 0.00 | 0.01 | 0.97 | 0.01 | 0.00 | 0.00 | 0.00 |
| cp5 | Tbb | "c" (blue) | 0.01 | 0.00 | 0.98 | 0.00 | 0.00 | 0.00 | 0.01 |
| cp14 | Tbb | "c" (blue) | 0.00 | 0.00 | 0.98 | 0.00 | 0.00 | 0.01 | 0.00 |
| OB58 | Tbb | uncertain | 0.00 | 0.40 | 0.00 | 0.45 | 0.01 | 0.12 | 0.01 |
| OB56 | Tbb | "d" (green) | 0.00 | 0.00 | 0.00 | 0.99 | 0.00 | 0.00 | 0.00 |
| OB54 | Tbr | "d" (green) | 0.00 | 0.00 | 0.00 | 0.99 | 0.00 | 0.00 | 0.00 |
| OB53 | Tbr | "d" (green) | 0.00 | 0.00 | 0.00 | 0.99 | 0.00 | 0.00 | 0.00 |
| OB57 | Tbr | "d" (green) | 0.00 | 0.00 | 0.00 | 0.98 | 0.01 | 0.00 | 0.00 |
| OB31 | Tbb | uncertain | 0.01 | 0.01 | 0.01 | 0.24 | 0.00 | 0.71 | 0.02 |
| OB10 | Tbb | uncertain | 0.00 | 0.00 | 0.00 | 0.44 | 0.00 | 0.54 | 0.00 |
| cp19 | Tbb | uncertain | 0.01 | 0.00 | 0.25 | 0.01 | 0.01 | 0.72 | 0.00 |
| OB027 | Tbr | uncertain | 0.00 | 0.00 | 0.26 | 0.01 | 0.03 | 0.60 | 0.10 |
| cp26 | Tbb | uncertain | 0.00 | 0.23 | 0.04 | 0.00 | 0.00 | 0.72 | 0.00 |
| cp7 | Tbb | uncertain | 0.00 | 0.18 | 0.01 | 0.00 | 0.00 | 0.79 | 0.01 |
| cp27 | Tbb | "f" (grey) | 0.00 | 0.02 | 0.05 | 0.00 | 0.00 | 0.92 | 0.01 |
| OB30 | Tbb | "f" (grey) | 0.02 | 0.00 | 0.12 | 0.01 | 0.00 | 0.84 | 0.01 |
| OB088 | Tbb | "f" (grey) | 0.00 | 0.00 | 0.00 | 0.01 | 0.00 | 0.98 | 0.00 |
| OB22 | Tbr | "f" (grey) | 0.01 | 0.00 | 0.00 | 0.01 | 0.00 | 0.97 | 0.00 |
| OB051 | Tbb | "f" (grey) | 0.00 | 0.01 | 0.01 | 0.01 | 0.00 | 0.97 | 0.00 |
| OB64 | Tbb | "f" (grey) | 0.01 | 0.03 | 0.00 | 0.02 | 0.01 | 0.92 | 0.02 |
| OB55 | Tbb | "f" (grey) | 0.02 | 0.01 | 0.00 | 0.01 | 0.00 | 0.96 | 0.01 |
| OB078 | Tbr | uncertain | 0.26 | 0.00 | 0.01 | 0.00 | 0.00 | 0.72 | 0.01 |
| OB066 | Tbr | uncertain | 0.00 | 0.34 | 0.00 | 0.00 | 0.01 | 0.63 | 0.01 |
| STIB366 | Tbb | uncertain | 0.25 | 0.22 | 0.00 | 0.01 | 0.01 | 0.50 | 0.01 |
| OB75 | Tbb | uncertain | 0.01 | 0.31 | 0.00 | 0.00 | 0.00 | 0.67 | 0.00 |
| OB65 | Tbr | uncertain | 0.00 | 0.56 | 0.00 | 0.00 | 0.01 | 0.41 | 0.01 |
| OB60 | Tbb | uncertain | 0.00 | 0.48 | 0.01 | 0.01 | 0.00 | 0.48 | 0.01 |
| OB153 | Tbb | "g" (red) | 0.00 | 0.00 | 0.01 | 0.00 | 0.00 | 0.00 | 0.98 |
| OB155 | Tbb | "g" (red) | 0.01 | 0.00 | 0.00 | 0.00 | 0.00 | 0.00 | 0.98 |
| OB095 | Tbr | "g" (red) | 0.01 | 0.00 | 0.01 | 0.00 | 0.02 | 0.01 | 0.96 |
| OB006 | Tbr | "g" (red) | 0.03 | 0.00 | 0.00 | 0.00 | 0.00 | 0.02 | 0.94 |
| OB113 | Tbb | "g" (red) | 0.01 | 0.02 | 0.01 | 0.00 | 0.01 | 0.05 | 0.89 |
| cp8 | Tbb | uncertain | 0.01 | 0.00 | 0.29 | 0.00 | 0.01 | 0.01 | 0.68 |
| OB026 | Tbr | "g" (red) | 0.00 | 0.00 | 0.01 | 0.01 | 0.02 | 0.01 | 0.94 |
| OB12 | Tbb | "g" (red) | 0.08 | 0.00 | 0.00 | 0.01 | 0.01 | 0.09 | 0.81 |
| OB024 | Tbr | uncertain | 0.01 | 0.00 | 0.37 | 0.00 | 0.01 | 0.01 | 0.59 |

| **B.** |  |  |  |  |  |  |  |  |  |  |
| --- | --- | --- | --- | --- | --- | --- | --- | --- | --- | --- |
| **Sample ID** | **RoTat 1.2** | **kDNA type** | **Genetic Cluster** | **“a”** | **“b”** | **“c”** | **“d”** | **“e”** | **“f”** | **“g”** |
| K2469 | - | A* | "e" (yellow) | 0.00 | 0.00 | 0.00 | 0.00 | 0.99 | 0.00 | 0.00 |
| K2444 | - | A* | "e" (yellow) | 0.00 | 0.00 | 0.00 | 0.00 | 0.99 | 0.00 | 0.00 |
| K2467 | - | A* | "e" (yellow) | 0.00 | 0.00 | 0.00 | 0.00 | 0.99 | 0.00 | 0.00 |
| K3789 | - | A* | "e" (yellow) | 0.00 | 0.00 | 0.00 | 0.00 | 0.99 | 0.00 | 0.00 |
| K3793 | - | Unkn. | "e" (yellow) | 0.01 | 0.00 | 0.00 | 0.00 | 0.96 | 0.00 | 0.02 |
| K3930 | - | A* | "e" (yellow) | 0.00 | 0.00 | 0.00 | 0.00 | 0.99 | 0.00 | 0.00 |
| K3931 | - | A* | "e" (yellow) | 0.00 | 0.00 | 0.00 | 0.00 | 0.99 | 0.00 | 0.00 |
| K2443 | - | A | "e" (yellow) | 0.00 | 0.00 | 0.00 | 0.00 | 0.99 | 0.00 | 0.00 |
| K2450 | - | Unkn. | "e" (yellow) | 0.01 | 0.00 | 0.00 | 0.01 | 0.97 | 0.00 | 0.01 |
| K2455 | - | A* | "e" (yellow) | 0.00 | 0.00 | 0.00 | 0.00 | 0.99 | 0.00 | 0.00 |
| K2458 | - | A* | "e" (yellow) | 0.00 | 0.00 | 0.00 | 0.00 | 0.99 | 0.00 | 0.00 |
| K2465 | - | A* | "e" (yellow) | 0.00 | 0.00 | 0.00 | 0.00 | 0.99 | 0.00 | 0.00 |
| K2466 | - | A* | "e" (yellow) | 0.00 | 0.00 | 0.00 | 0.00 | 0.99 | 0.00 | 0.00 |
| K2470 | - | A* | "e" (yellow) | 0.00 | 0.00 | 0.00 | 0.00 | 0.99 | 0.00 | 0.00 |
| K2439 | + | A | "e" (yellow) | 0.00 | 0.03 | 0.01 | 0.01 | 0.94 | 0.01 | 0.00 |
| K2441 | + | A* | "e" (yellow) | 0.00 | 0.00 | 0.00 | 0.00 | 0.99 | 0.00 | 0.00 |
| K2442 | + | A* | "e" (yellow) | 0.00 | 0.00 | 0.00 | 0.00 | 0.99 | 0.00 | 0.00 |
| K2446 | + | A* | "e" (yellow) | 0.00 | 0.00 | 0.00 | 0.00 | 0.99 | 0.00 | 0.00 |
| K2449 | + | A* | "e" (yellow) | 0.00 | 0.00 | 0.00 | 0.00 | 0.99 | 0.00 | 0.00 |
| K2451 | + | A* | "e" (yellow) | 0.00 | 0.00 | 0.00 | 0.00 | 0.99 | 0.00 | 0.00 |
| K2453 | + | A* | "e" (yellow) | 0.00 | 0.00 | 0.00 | 0.00 | 0.98 | 0.01 | 0.01 |
| K2454 | + | A | "e" (yellow) | 0.00 | 0.00 | 0.00 | 0.00 | 0.99 | 0.00 | 0.00 |
| K2456 | + | A | "e" (yellow) | 0.00 | 0.00 | 0.00 | 0.00 | 0.99 | 0.00 | 0.00 |
| K2457 | + | A* | "e" (yellow) | 0.00 | 0.00 | 0.00 | 0.00 | 0.99 | 0.00 | 0.00 |
| K2479 | - | B | "f" (grey) | 0.01 | 0.00 | 0.01 | 0.01 | 0.01 | 0.93 | 0.03 |
| K2481 | + | A* | "e" (yellow) | 0.00 | 0.00 | 0.00 | 0.00 | 0.99 | 0.00 | 0.00 |
| K3548 | + | A* | "e" (yellow) | 0.00 | 0.00 | 0.00 | 0.00 | 0.99 | 0.00 | 0.00 |
| K3550 | + | A* | "e" (yellow) | 0.00 | 0.00 | 0.00 | 0.00 | 0.99 | 0.00 | 0.00 |
| K3551 | + | A* | "e" (yellow) | 0.00 | 0.00 | 0.00 | 0.00 | 0.99 | 0.00 | 0.00 |
| K3552 | + | non-A/B | "c" (blue) | 0.00 | 0.00 | 0.99 | 0.00 | 0.00 | 0.00 | 0.00 |
| K3553 | + | A* | "e" (yellow) | 0.04 | 0.00 | 0.00 | 0.00 | 0.95 | 0.00 | 0.00 |
| K3556 | + | A* | "e" (yellow) | 0.00 | 0.00 | 0.00 | 0.00 | 0.99 | 0.00 | 0.00 |
| K3557 | + | non-A/B | "c" (blue) | 0.00 | 0.00 | 0.99 | 0.00 | 0.00 | 0.00 | 0.00 |
| K3558 | + | A* | "e" (yellow) | 0.04 | 0.00 | 0.00 | 0.00 | 0.95 | 0.00 | 0.00 |
| K3576 | + | Unkn. | "e" (yellow) | 0.10 | 0.00 | 0.00 | 0.00 | 0.87 | 0.01 | 0.02 |
| STIB810 | + | A | uncertain | 0.02 | 0.03 | 0.03 | 0.02 | 0.65 | 0.03 | 0.22 |
| C13 | + | A | "e" (yellow) | 0.06 | 0.01 | 0.00 | 0.01 | 0.86 | 0.04 | 0.02 |
| RoTat1.2 (OB106) | + | A | "g" (red) | 0.08 | 0.02 | 0.00 | 0.00 | 0.08 | 0.00 | 0.81 |
| STIB708 (OB35) | + | A | uncertain | 0.01 | 0.00 | 0.01 | 0.02 | 0.16 | 0.01 | 0.78 |
| STIB806K (OB2) | + | A | uncertain | 0.01 | 0.01 | 0.02 | 0.00 | 0.39 | 0.01 | 0.57 |
| STIB811 (OB42) | + | A | "g" (red) | 0.00 | 0.01 | 0.00 | 0.00 | 0.04 | 0.00 | 0.94 |

* kDNA type is tentative because it is based on the A281del PCR assay only.
