# Supplementary material for: Multiple evolutionary origins of Trypanosoma evansi in Kenya
Source: PLoS Negl Trop Dis. 2017 Sep 7;11(9):e0005895. doi: 10.1371/journal.pntd.0005895 (PMC5605091; doi:10.1371/journal.pntd.0005895)
Supplement: S4 Table — Within-cluster distance using STRUCTURE-based [51] genetic clusters including strains with Q values > 0.80 (S3 Table) for (A) all strains regardless of taxonomy, (B) T. brucei (Tb) strains, and (C) T. evansi (Tev) strains. Number of pairwise between-strain comparisons (N pairs), mean Reynolds (1983) [56] distance (mean distance) estimated in the R package “PopPR” v2.3.0 [57, 58], standard deviation (SD), minimum distance (min), and maximum distance (max). (DOCX) [file pntd.0005895.s009.docx]

**S4 Table.** Within-cluster distance using STRUCTURE-based [51] genetic clusters including strains with Q values > 0.80 (Table S3) for **(A)** all strains regardless of taxonomy, **(B)** *T. brucei* (Tb) strains, and **(C)** *T. evansi* (Tev) strains. Number of pairwise between-strain comparisons (N pairs), mean Reynolds (1983) [56] distance (mean distance) estimated in the R package “PopPR” *v2.3.0* [57, 58], standard deviation (SD), minimum distance (min), and maximum distance (max).

| **A.** | **N pairs** | **Mean distance** | **SD** | **Min** | **Max** |
| --- | --- | --- | --- | --- | --- |
| “a” (orange) | 28 | 0.67 | 0.15 | 0.37 | 0.82 |
| “b” (purple) | 240 | 0.73 | 0.06 | 0.27 | 0.84 |
| “c” (blue) | 90 | 0.74 | 0.09 | 0.22 | 0.85 |
| “d” (green) | 12 | 0.61 | 0.11 | 0.44 | 0.72 |
| “e” (yellow) | 1188 | 0.57 | 0.13 | 0.21 | 0.77 |
| “f” (grey) | 56 | 0.80 | 0.06 | 0.71 | 0.90 |
| “g” (red) | 72 | 0.78 | 0.05 | 0.57 | 0.85 |
| **Overall** | **1686** | **0.70** | **0.10** | **0.21** | **0.90** |
|  |  |  |  |  |  |
| **B.** | **N pairs** | **Mean distance** | **SD** | **Min** | **Max** |
| Tb “a” (orange) | 28 | 0.67 | 0.15 | 0.37 | 0.82 |
| Tb “b” (purple) | 240 | 0.73 | 0.06 | 0.27 | 0.84 |
| Tb “c” (blue) | 56 | 0.76 | 0.06 | 0.65 | 0.85 |
| Tb “d” (green) | 12 | 0.61 | 0.11 | 0.44 | 0.72 |
| Tb “f” (grey) | 42 | 0.81 | 0.06 | 0.71 | 0.90 |
| Tb “g” (red) | 30 | 0.76 | 0.06 | 0.57 | 0.82 |
| **Overall Tb** | **408** | **0.72** | **0.08** | **0.27** | **0.90** |
|  |  |  |  |  |  |
| **C.** | **N pairs** | **Mean distance** | **SD** | **Min** | **Max** |
| Tev “c/f” (blue/grey) | 6 | 0.60 | 0.29 | 0.22 | 0.78 |
| Tev “e” (yellow) | 1188 | 0.57 | 0.13 | 0.21 | 0.77 |
| Tev “g” (red) | 6 | 0.75 | 0.01 | 0.74 | 0.75 |
| **Overall Tev** | **1200** | **0.64** | **0.14** | **0.21** | **0.78** |
