# Supplementary material for: Multiple evolutionary origins of Trypanosoma evansi in Kenya
Source: PLoS Negl Trop Dis. 2017 Sep 7;11(9):e0005895. doi: 10.1371/journal.pntd.0005895 (PMC5605091; doi:10.1371/journal.pntd.0005895)
Supplement: S5 Table — Summary of differences in within-cluster Reynolds [56] distance of STRUCTURE-defined clusters based on analysis of variance (ANOVA, p-value < 0.0001), and the Tukey-Kramer HSD test performed in JMP v11.2 (SAS Institute Inc., Cary, NC, USA, 1989–2012), using only the 86 strains with Q values >0.80 (S3 Table): (A) Ordered difference report between clusters showing the clusters compared (cluster 1 and cluster 2), the difference in mean Reynolds distance (Dif), the standard error of the difference (Std Err Dif), the lower confidence level (CL), the upper confidence level (CL), and the p-value of the pairwise comparison. (B) The connecting symbols report that summarizes the Tukey-Kramer HSD tests, where each symbol group (¥, §, †, •, *) contain significantly different within-cluster pairwise genetic distances (¥ joins clusters “d” and “e”, § joins clusters “a” and “d”; † joins clusters “a”, “b”, and “c”; • joins clusters “a”, “b”, “c”, and “g”; and * joins clusters “c”, “f”, and “g”). (DOCX) [file pntd.0005895.s010.docx]

**S5 Table.** Summary of differences in within-cluster Reynolds [56] distance of STRUCTURE-defined clusters based on analysis of variance (ANOVA, p-value < 0.0001), and the Tukey-Kramer HSD test performed in JMP v11.2 (SAS Institute Inc., Cary, NC, USA, 1989– 2012), using only the 86 strains with Q values >0.80 (Table S3): **(A)** Ordered difference report between clusters showing the clusters compared (cluster 1 and cluster 2), the difference in mean Reynolds distance (Dif), the standard error of the difference (Std Err Dif), the lower confidence level (CL), the upper confidence level (CL), and the p-value of the pairwise comparison. **(B)** The connecting symbols report that summarizes the Tukey-Kramer HSD tests, where each symbol group (¥, §, †, •, *) contain significantly different within-cluster pairwise genetic distances (¥ joins clusters “d” and “e”, § joins clusters “a” and “d”; † joins clusters “a”, “b”, and “c”; • joins clusters “a”, “b”, “c”, and “g”; and * joins clusters “c”, “f”, and “g”).

| **A.** |  |  |  |  |  |  |
| --- | --- | --- | --- | --- | --- | --- |
| **Cluster 1** | **Cluster 2** | **Dif** | **Std Err Dif** | **Lower CL** | **Upper CL** | **p-Value** |
| within “f” | within “e” | 0.24 | 0.02 | 0.18 | 0.29 | <.0001 |
| within “f” | within “d” | 0.20 | 0.04 | 0.08 | 0.31 | <.0001 |
| within “g” | within “e” | 0.19 | 0.02 | 0.13 | 0.25 | <.0001 |
| within “c” | within “e” | 0.17 | 0.02 | 0.12 | 0.21 | <.0001 |
| within “b” | within “e” | 0.16 | 0.01 | 0.13 | 0.18 | <.0001 |
| within “g” | within “d” | 0.15 | 0.04 | 0.03 | 0.27 | 0.003 |
| within “f” | within “a” | 0.14 | 0.03 | 0.05 | 0.23 | <.0001 |
| within “c” | within “d” | 0.13 | 0.04 | 0.02 | 0.24 | 0.010 |
| within “b” | within “d” | 0.12 | 0.04 | 0.02 | 0.22 | 0.010 |
| within “a” | within “e” | 0.10 | 0.02 | 0.03 | 0.16 | 0.001 |
| within “g” | within “a” | 0.09 | 0.03 | 0.00 | 0.18 | 0.038 |
| within “f” | within “b” | 0.08 | 0.02 | 0.02 | 0.14 | 0.002 |
| within “c” | within “a” | 0.07 | 0.03 | -0.01 | 0.15 | 0.137 |
| within “f” | within “c” | 0.07 | 0.02 | 0.00 | 0.14 | 0.060 |
| within “b” | within “a” | 0.06 | 0.02 | -0.01 | 0.13 | 0.133 |
| within “a” | within “d” | 0.06 | 0.04 | -0.06 | 0.18 | 0.764 |
| within “f” | within “g” | 0.05 | 0.03 | -0.03 | 0.13 | 0.550 |
| within “d” | within “e” | 0.04 | 0.03 | -0.07 | 0.14 | 0.944 |
| within “g” | within “b” | 0.03 | 0.02 | -0.03 | 0.09 | 0.794 |
| within “g” | within “c” | 0.02 | 0.03 | -0.05 | 0.10 | 0.981 |
| within “c” | within “b” | 0.01 | 0.02 | -0.04 | 0.06 | 0.999 |
|  |  |  |  |  |  |  |
| **B** |  |  |  |  |  |  |
| **Cluster** | **Group "*"** | **Group "•"** | **Group**  **"†"** | **Group "§"** | **Group "¥"** |  |
| within “a” |  |  | † | § |  |  |
| within “b” |  | • | † |  |  |  |
| within “c” | * | • | † |  |  |  |
| within “d” | |  |  | § | ¥ |  |
| within “e” |  |  |  |  | ¥ |  |
| within “f” | * |  |  |  |  |  |
| within “g” | * | • |  |  |  |  |
