# Supplementary material for: Multiple evolutionary origins of Trypanosoma evansi in Kenya
Source: PLoS Negl Trop Dis. 2017 Sep 7;11(9):e0005895. doi: 10.1371/journal.pntd.0005895 (PMC5605091; doi:10.1371/journal.pntd.0005895)
Supplement: S6 Table — Among-cluster genetic differentiation (FST) among each STRUCTURE-defined [51] genetic cluster, using only strains with Q values >0.80 (S3 Table): (A) all strains, (B) T. brucei (Tb) strains only, and (C) T. evansi (Tev) strains only. Pairwise FST (below diagonal) was calculated in ARLEQUIN v.3.2 [59] with Wright’s statistics [60], following the variance method developed by Weir and Cockerham (1984) [61] using 10,000 permutations to obtain exact p-values (above diagonal), with the only non-significant FST found (between T. evansi cluster “e” and “g”) in bold. (DOCX) [file pntd.0005895.s011.docx]

**S6 Table.** Among-cluster genetic differentiation (F_ST_) among each STRUCTURE-defined [51] genetic cluster, using only strains with Q values >0.80 (Tab. S3): **(A)** all strains, **(B)** *T. brucei* (Tb) strains only, and **(C)** *T. evansi* (Tev) strains only. Pairwise F_ST_ (below diagonal) was calculated in ARLEQUIN *v.3.2* [59] with Wright’s statistics [60], following the variance method developed by Weir and Cockerham (1984) [61] using 10,000 permutations to obtain exact p-values (above diagonal), with the only non-significant F_ST_ found (between *T. evansi* cluster “e” and “g”) in bold.

| **A.** | "a" | "b" | "c" | "d" | "e" | "f" | "g" |  |  |  |
| --- | --- | --- | --- | --- | --- | --- | --- | --- | --- | --- |
| "a" |  | 0.000 | 0.000 | 0.006 | 0.000 | 0.000 | 0.000 |  |  |  |
| "b" | 0.21 |  | 0.000 | 0.000 | 0.000 | 0.000 | 0.000 |  |  |  |
| "c" | 0.17 | 0.16 |  | 0.001 | 0.000 | 0.000 | 0.000 |  |  |  |
| "d" | 0.31 | 0.18 | 0.18 |  | 0.000 | 0.002 | 0.001 |  |  |  |
| "e" | 0.11 | 0.23 | 0.15 | 0.14 |  | 0.000 | 0.000 |  |  |  |
| "f" | 0.15 | 0.15 | 0.10 | 0.16 | 0.12 |  | 0.000 |  |  |  |
| "g" | 0.13 | 0.17 | 0.13 | 0.20 | 0.08 | 0.11 |  |  |  |  |
|  |  |  |  |  |  |  |  |  |  |  |
| **B.** | Tb “a” | Tb “b” | Tb “c” | Tb “d” | Tb “f” | Tb “f” |  |  |  |  |
| Tb “a” |  | 0.000 | 0.001 | 0.005 | 0.001 | 0.002 |  |  |  |  |
| Tb “b” | 0.21 |  | 0.000 | 0.000 | 0.000 | 0.000 |  |  |  |  |
| Tb “c” | 0.18 | 0.15 |  | 0.003 | 0.001 | 0.000 |  |  |  |  |
| Tb “d” | 0.31 | 0.18 | 0.19 |  | 0.004 | 0.005 |  |  |  |  |
| Tb “f” | 0.16 | 0.15 | 0.10 | 0.17 |  | 0.001 |  |  |  |  |
| Tb “g” | 0.15 | 0.17 | 0.15 | 0.27 | 0.14 |  |  |  |  |  |
|  |  |  |  |  |  |  |  |  |  |  |
| **C.** | Tev “c/f” | Tev “e” | Tev “g” |  |  |  |  |  |  |  |
| Tev “c/f” |  | 0.000 | **0.105** |  |  |  |  |  |  |  |
| Tev “e” | 0.29 |  | 0.000 |  |  |  |  |  |  |  |
| Tev “g” | **0.06** | 0.09 |  |  |  |  |  |  |  |  |
